# High-Performance Semiconducting Carbon Nanotube Transistors Using Naphthalene Diimide-Based Polymers with Biaxially Extended Conjugated Side Chains

**DOI:** 10.1021/acsami.4c08981

**Published:** 2024-08-13

**Authors:** Chun-Chi Chen, Shang-Wen Su, Yi-Hsuan Tung, Po-Yuan Wang, Sheng-Sheng Yu, Chi-Cheng Chiu, Chien-Chung Shih, Yan-Cheng Lin

**Affiliations:** † Department of Chemical Engineering, National Cheng Kung University, Tainan 70101, Taiwan; ‡ Advanced Research Center for Green Materials Science and Technology, National Taiwan University, Taipei 10617, Taiwan; § Department of Chemical Engineering and Materials Engineering, National Yunlin University of Science and Technology, Douliou, Yunlin 64002, Taiwan

**Keywords:** biaxial conjugation, conjugated polymers, single-walled carbon nanotubes, sorting, field-effect transistors

## Abstract

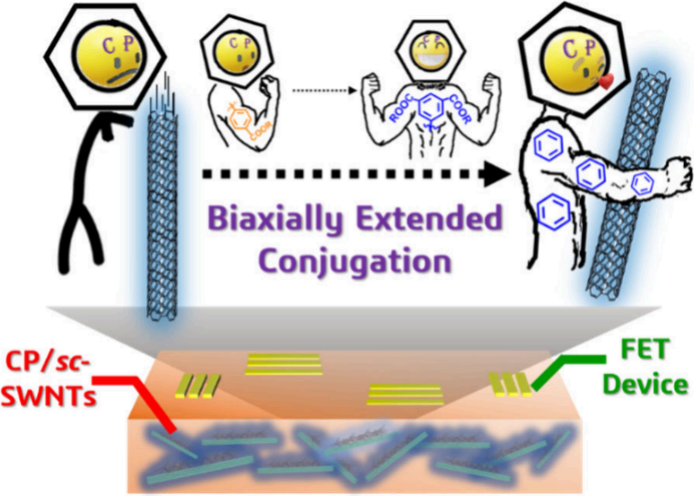

Polymer-wrapped single-walled carbon nanotubes (SWNTs) are a potential method for obtaining high-purity semiconducting (*sc*) SWNT solutions. Conjugated polymers (CPs) can selectively sort *sc*-SWNTs with different chiralities, and the structure of the polymer side chains influences this sorting capability. While extensive research has been conducted on modifying the physical, optical, and electrical properties of CPs through side-chain modifications, the impact of these modifications on the sorting efficiency of *sc*-SWNTs remains underexplored. This study investigates the introduction of various conjugated side chains into naphthalene diimide-based CPs to create a biaxially extended conjugation pattern. The CP with a branched conjugated side chain (**P3**) exhibits reduced aggregation, resulting in improved wrapping ability and the formation of larger bundles of high-purity *sc*-SWNTs. Grazing incidence X-ray diffraction analysis confirms that the potential interaction between *sc*-SWNTs and CPs occurs through π–π stacking. The field-effect transistor device fabricated with **P3**/*sc*-SWNTs demonstrates exceptional performance, with a significantly enhanced hole mobility of 4.72 cm^2^ V^–1^ s^–1^ and high endurance/bias stability. These findings suggest that biaxially extended side-chain modification is a promising strategy for improving the sorting efficiency and performance of *sc*-SWNTs by using CPs. This achievement can facilitate the development of more efficient and stable electronic devices.

## Introduction

Conjugated polymers (CPs) have been widely applied to organic optoelectronic devices because of their malleability, self–assembly properties, solution processability, large-area flexibility, eco-friendliness, and excellent compatibility with stretchable electronics.^1−3^ Organic field-effect transistors (FETs) can be diversely functionalized and further improved owing to the ability to perform structural modifications of organic materials.^4−6^ Recent studies have demonstrated that side-chain modifications such as biaxial conjugated extension on CPs could modify their backbone coplanarity, chain packing patterns, and optical absorptions.^7−9^ In addition, the biaxial conjugated extension can improve the charge-transport performance and mobility–stretchability of CPs.^10−14^ However, the application of the biaxial conjugated extension in N-type CPs is undeveloped. Recently, naphthalene diimide (NDI)-based CPs have been intensively investigated.^15,16^ Therefore, improving the performance of NDI-based CPs using side-chain engineering is of great importance and research interest.^17^

The research interest in single-walled carbon nanotubes (SWNTs) persists due to their remarkable mechanical and electrical properties, positioning them as promising candidates for various advanced applications. SWNTs are being explored for integration into FETs, thermoelectric devices, biosensors, and solar cells, which could serve as active layers and significantly enhance the device’s performance.^18^ Nevertheless, commercially available SWNTs contain one-third metallic SWNTs (*m*-SWNTs), two-thirds semiconducting SWNTs (*sc*-SWNTs), some amorphous carbon, and catalysts for the rest. In this case, the purification and sorting of *sc*-SWNTs become crucial for fabricating high-performance devices. Based on previous literature, the *sc*-SWNTs could be sorted by using the following approaches: (i) density gradient ultracentrifugation,^19,20^ (ii) gel agarose chromatography,^21^ and (iii) noncovalent bond selective wrapping of *sc*-SWNTs with DNA^22^ and CPs.^23−25^ From a scale-up point of view, noncovalent bond selective wrapping is preferable. From an economic point of view, the last approach above, wrapping by DNA, is too expensive to apply, so CPs are the potential choice. With regard to the backbone engineering of CP, Lei et al. developed fluorene-based polyazomethine as a removable and recyclable CP for highly selective and high-yield dispersion and release of low-cost *sc*-SWNTs.^23^ Hwang et al. used a series of diketopyrrolopyrrole (DPP)-based CPs for effectively enriching *sc*-SWNTs of the high-pressure carbon monoxide (HiPco) SWNTs and obtaining high-quality *sc*-SWNT solutions without impurities due to the dispersibility from the slightly kinked backbone.^24^

With regard to the side-chain modification of CPs, Gomulya et al. investigated the side-chain length effect of polyfluorene in *sc*-SWNT sorting. They found that CPs with long alkyl side chains can wrap *sc*-SWNTs with large diameters and chirality because of their different configurations, with the CP backbone perpendicular (T) or parallel (P) to the tube surface. This is the first research on side-chain modification of CPs in *sc*-SWNT sorting.^25^ Later, Wang et al. used a polyfluorene-based alternative copolymer modified with a benzophenone group for wrapping and achieving higher yield than the reference CP of poly(9,9-dioctylfluorene) via a solution process.^26^ Then, they exposed the CP/*sc*-SWNT film to UV irradiation to pattern the film through photolithography. Recently, Ye and Talsma et al. used NDI-based CPs with low bandgap and polar/nonpolar side chains for wrapping, and they found that adjusting side chains could influence dispersion at a certain level.^27,28^ Ouyang et al. reported a backbone strategy of polyfluorene with C–C or C=C linkages for wrapping different chiralities of *sc*-SWNTs. They found that, through multicycle conjugated polymer extraction processes, the single chirality *sc*-SWNTs could be enriched and show better purity, yield, and selectivity. This can certainly improve the performance of thin-film transistors.^29^ Luo et al. showed another backbone strategy using pentiptycene polymers containing metal-chelating groups for immortalizing metal selectors on *sc*-SWNT chemiresistors. They can serve as breath biomarkers, improving the life of patients suffering from chronic kidney disease.^30^ Previously, we developed a series of NDI-based CPs with different donors to sort *sc*-SWNTs for advanced phototransistor memory.^31^ The result indicated that the CP’s coplanarity and aggregation play an important role in the wrapping of *sc*-SWNTs. In addition, the design concept of extractor and enhancer with a synergistic effect is also practical in *sc*-SWNT sorting.^32^ Nonetheless, no research has systematically investigated the influence of side-chain modification, especially the biaxially extended conjugations of CPs on *sc*-SWNT sorting. The conjugated side chain in biaxially extended conjugations of CPs can enlarge the side chain domain to potentially optimize the wrapping patterns on *sc*-SWNTs.

There is limited research on the relationships between side-chain modification of CPs and *sc*-SWNT sorting thus far, and there is no study on the influence of biaxially extended conjugation in NDI-based CPs. Therefore, in this study, we modified NDI-based n-type CPs for wrapping *sc*-SWNTs with conjugated side chains. By introducing conjugated side chains into the CPs, we explored the relationship between side-chain modifications and wrapping of *sc*-SWNTs. Accordingly, the conventional CP with a donor of bithiophene (2T) and an alkyl side chain is named **P1**. The CPs with conjugated dibenzoyl imide moieties and linear or branched alkyl side chains are named **P2** and **P3**. These NDI-based CPs showed good selectivity to plasma discharge SWNTs (PD-SWNTs). The sorting efficiency of CP/*sc*-SWNT solutions was evaluated using ultraviolet–visible–near-infrared (UV–vis–NIR) and Raman spectroscopies. The morphology of the CP/*sc*-SWNT films was probed using atomic force microscopy (AFM) and grazing incidence X-ray diffraction (GIXD) techniques. We surprisingly discovered that the noncovalent bond interactions between CP and *sc*-SWNT, which is a significant way for wrapping *sc*-SWNTs, might be influenced by the size and branches of the conjugated side chains in CPs. The experimental results are consistent with the calculated density functional theory (DFT) and molecular dynamics (MD) simulations. Furthermore, we found that the device of **P3**/*sc*-SWNTs demonstrated better electrical performance: the mobility of the device increased to 4.72 cm^2^ V^–1^ s^–1^ of **P3**-wrapped *sc*-SWNTs, which is significantly higher than that of 2.21 cm^2^ V^–1^ s^–1^ for **P1**-wrapped *sc*-SWNTs. In addition, the device of **P3**/*sc*-SWNTs owns a higher endurance stability. Therefore, the NDI-based CPs with biaxially extended side chains enhance the wrapping selectivity of *sc*-SWNTs to improve the FET device performance.

## Results and Discussion

### Synthesis, Optical, and Electrochemical Characterizations of the Polymers

**Scheme 1:**
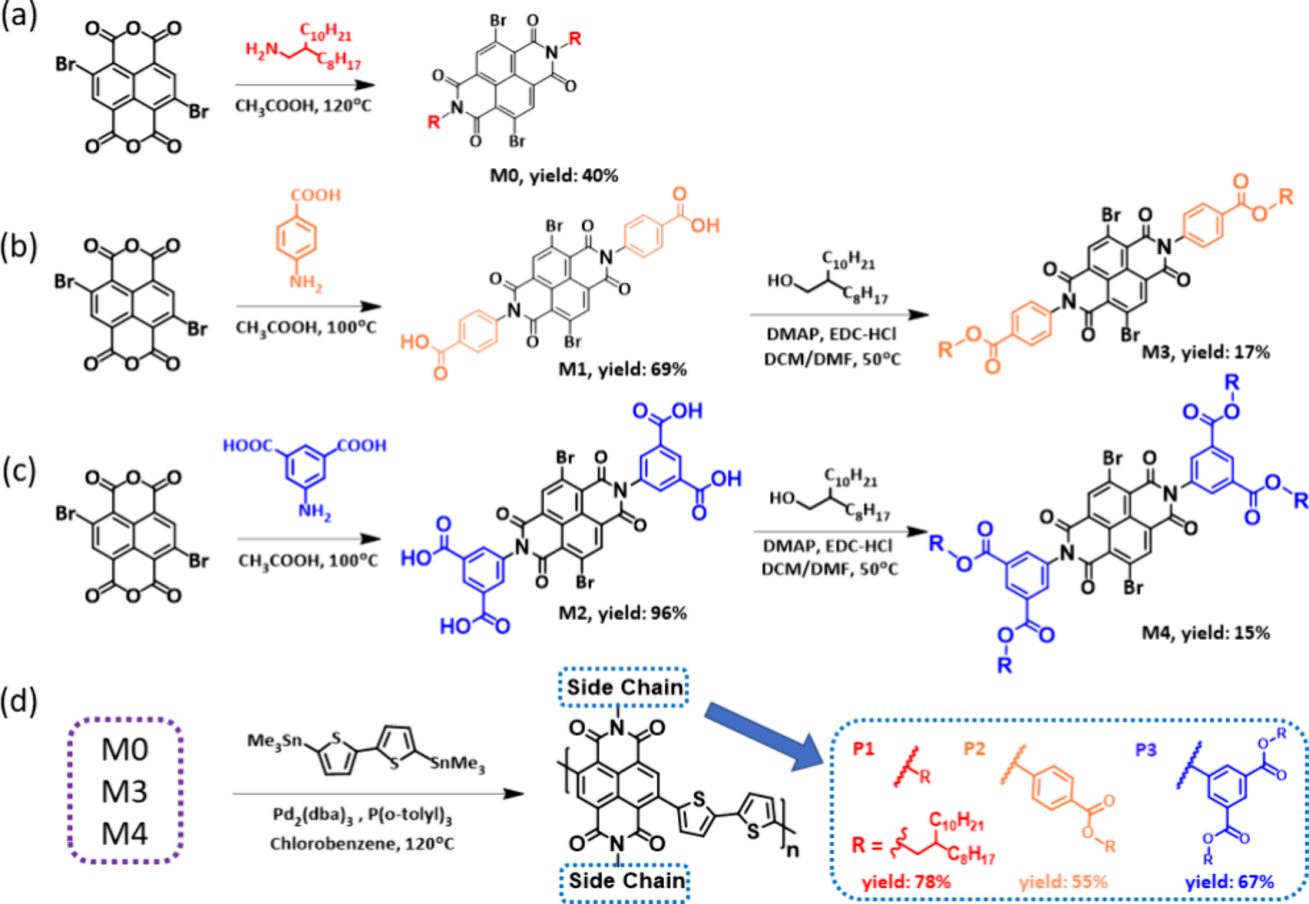
Synthetic Routes for the Naphthalene Diimide Monomers with the (a) Alkyl Groups, (b) Alkyl Benzoate Groups, (c) Di-Alkyl Benzoate Groups, and (d) Polymerization of CPs with Branched and Conjugated Side Chain Moieties

**Figure 1:**
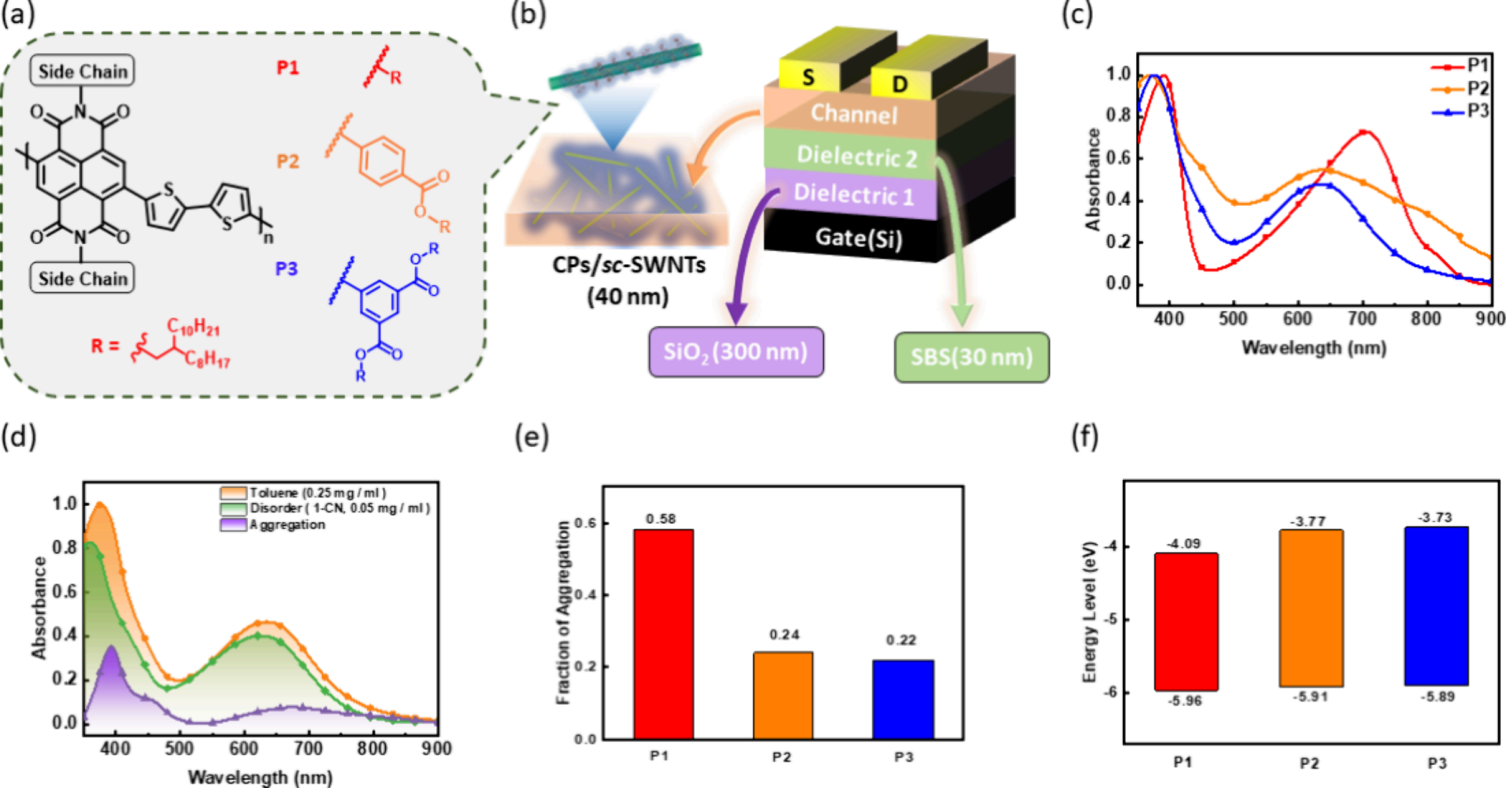
(a) Chemical structures of the reported NDI-based CPs. (b) Device structure with a semiconducting channel composed of CP/*sc*-SWNT. (c) UV–vis absorption spectra of the pure CP solutions. (d) Fractions of aggregate and disorder in the UV–vis absorption spectra of **P3** solution. (e) Fractions of the aggregate evaluated based on the UV–vis absorption spectra of CP solutions in toluene and in 1-chloronaphthalene. (f) Frontier energy levels of the CPs studied.

Wrapping *sc-*SWNTs via CPs through sorting processes has been broadly considered a high-efficiency way for fabricating *sc*-SWNTs-based FET devices. Modified CPs with conjugated side chains enlarge the polymers’ size and modify their characteristics. Several properties, such as physical, optical, electrochemical, and aggregation behavior, have changed by extending the molecule’s size. Figure 1a displays a series of NDI-based CPs synthesized in this study; their synthetic routes, chemical structures, and monomers are shown in Scheme 1. **M1** was synthesized with a benzoic acid side group by imidization from brominated naphthalene diamide (Br–NDA–Br) and alkylated with octyl-dodecane by Steglich esterification for obtaining **M3**.^33^**M2** was synthesized with 5-amino isophthalic acid and Br–NDA–Br by imidization, and **M4** was obtained by alkylation with octyl-dodecane. The phenyl group modified NDI-based monomers were polymerized through Pd^(0)^-catalyzed Migita–Kosugi–Stille coupling polymerization with bithiophene.^34^ More detailed steps for synthesis are presented in the Experimental Section. Figure S1 demonstrates the gel permeation chromatography (GPC) profiles with THF as an eluent. The monomers’ ^1^H and ^13^C NMR spectra are shown in Figures S2–S9 (Supporting Information), and the polymers’ ^1^H and ^13^C NMR spectra are shown in Figures S10–S15 (Supporting Information). The chemical shifts and integral areas correspond to each chemical structure. Regarding the thermal properties, the decomposition temperature (*T*_d_) and glass transition temperature (*T*_g_) measured by the thermogravimetric analyzer (TGA) and differential scanning calorimetry (DSC) are shown in Figure S16 (Supporting Information). The *T*_g_ of **P3** can be observed at 170 °C because it contains more alkyl side chains, making the polymer softer than those of **P1** and **P2**. As summarized in Table S1 (Supporting Information), the *T*_d_ values of these CPs are high and at 436, 312, and 358 °C, respectively, representing their high thermal stabilities.

The CPs were applied to wrap and sort the *sc*-SWNTs selectively. The composite was used as the semiconducting channel for FET devices, including the thickness of the dielectric and channel layers (Figure 1b). Table S2 shows the device information, including the layer thickness and dielectric properties. Different sizes of side chains might induce intrachain charge transfer (ICT) related to phenyl groups, chain coplanarities, and steric hindrances, primarily owing to the long alkyl chains. Figure 1c reveals the UV–vis–NIR absorption spectrum of the CPs studied. The (0:1) peaks of **P1**, **P2**, and **P3** are at 705, 637, and 635 nm, respectively. In addition, **P1**’s π–π* peak is at 391 nm, while **P2** and **P3**’s peaks blue shift to 368 and 375 nm. It might be caused by reducing the density of electrons and backbone aggregations. Figure 1d represents **P3**’s aggregation behavior, and the rest of **P1** and **P2** is shown in Figure S17 (Supporting Information). Note that the polymer solutions in toluene were prepared at a concentration of 0.25 mg mL^–1^, the same concentration in the sorting solution discussed in the following sections; the disordered state was the polymer solution prepared in 1-chloronaphthalene at a concentration of 0.05 mg mL^–1^. The calculation is detailed in the Experimental Section. The calculated aggregation fraction of the CPs is shown in Figure 1e. Accordingly, the aggregation fractions are 0.58, 0.24, and 0.22 for **P1**, **P2**, and **P3**, respectively. **P1** had the highest value, while **P2** and **P3** had values lower than those of **P1**. It might be attributed to side chain modifications and the hindrance of the long/branched alkyl chain. Compared with **P1**, CPs with phenyl side groups are more sterically hindered in the solvent, resulting in a relatively lower aggregation fraction. Furthermore, the lower aggregation fraction of **P3** than **P2** is because the branched side chains dilute the contents and interactions of main chains so that **P3** would be more dispersed in the solvent.^35^ To summarize, conjugated side-chain extension with such phenyl groups has a particular intrinsic impact on the aggregation properties of the polymers. This outcome can also have a significant influence on the sorting processes.

Figure 1f and Table S1 (Supporting Information) present the energy level of each polymer calculated by UV–vis–NIR spectroscopy. The lowest unoccupied molecular orbital (LUMO) level was calculated from the cyclic voltammetry profiles (CV, Figure S18, Supporting Information): LUMO = −*e*·(*E*_re_ – *E*_1/2_(ferrocene) + 4.8), where LUMO is the lowest unoccupied molecular orbital, *e* is the elementary charge, and *E*_re_ represents the reduction potential in the CV sweeping. The calculation of the energy bandgap is calculated by *E*_g_ = 1240/λ_onset_, where λ_onset_ is the onset wavelength. The calculation of HOMO is HOMO = LUMO – *E*_g_, where HOMO is the highest occupied molecular orbital. As **P2** and **P3** blue-shift to 635 nm in the (0–1) peak, the LUMO decreased to −3.77 and −3.73 eV, respectively. The conjugated side-chain extension with phenyl groups makes an increase in the energy levels. It might be attributed to the improved structural rigidity by the biaxial extension of the benzene rings. The conjugated side groups may disrupt the delocalization of the electrons on NDI acceptors along the backbone, thus enabling increased LUMO levels.^36^ From the above calculations, **P2** and **P3** are polymers with a higher electron-donating ability that might possess higher ICT intrinsically.^37^ However, the branched alkyl side chains of **P3** hinder its aggregation, thereby showing a more blue-shifted ICT band.

### Polymer/*sc*-SWNT Sorting Properties

**Figure 2:**
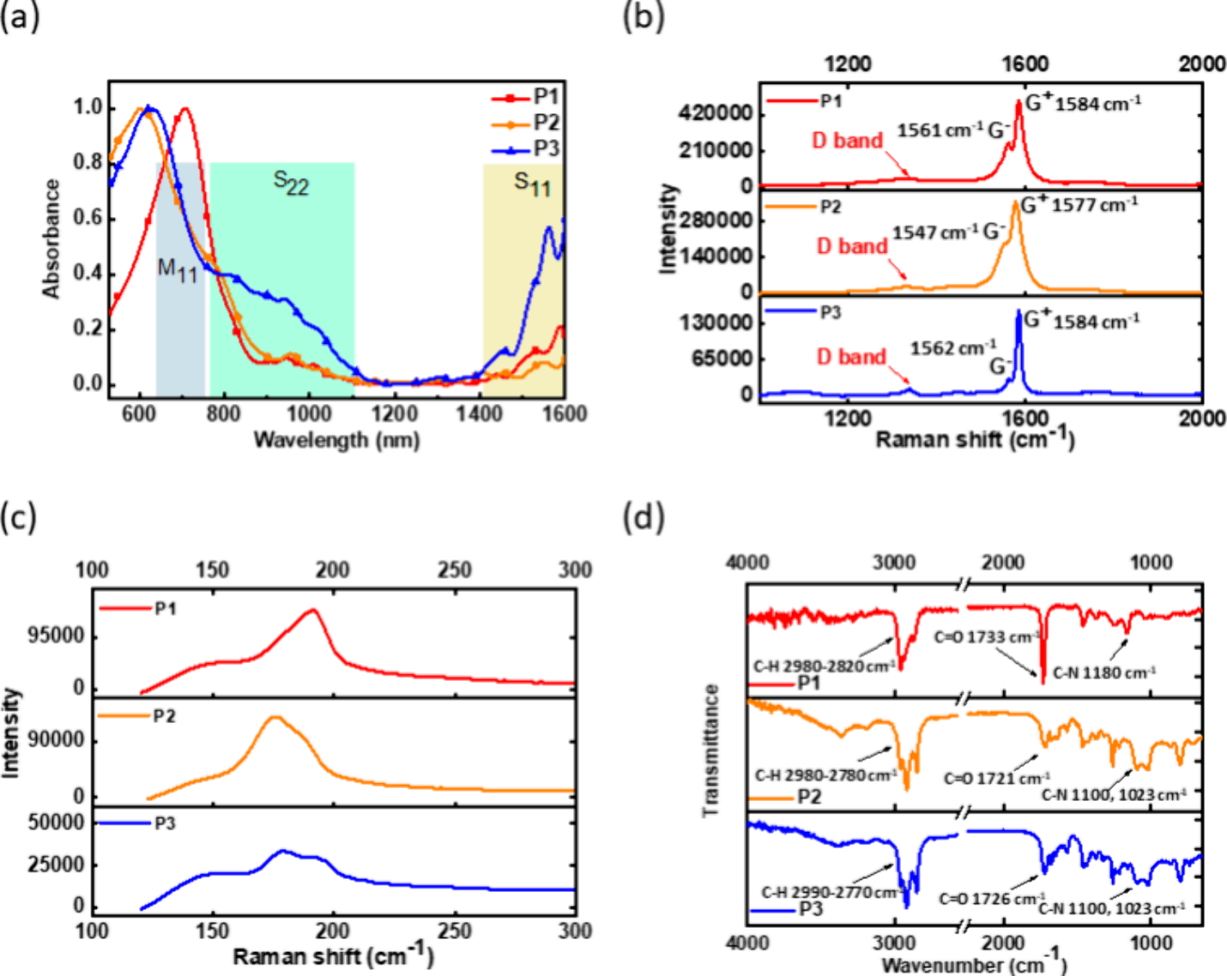
(a) UV–vis absorption spectra of the CP/*sc*-SWNT sorting solutions. Raman spectra of the drop-cast CP/*sc*-SWNT films with an excitation wavelength of 633 nm at the bands spanning the range of (b) 1000 to 2000 cm^–1^ and (c) 100 to 300 cm^–1^. (d) FT–IR spectra of the CP/*s*-SWNT films.

The CPs and PD-SWNTs were sonicated in toluene with a bar sonicator to sort the *sc*-SWNTs. After sonication, the mixed solution with sufficiently dispersed CPs entangled with *sc*-SWNTs was centrifugated to remove impurities, like amorphous carbon and *m*-SWNTs. The UV–vis–NIR absorption characterization was conducted to gain more insight into the sorting solutions, and the results are displayed in Figure 2a. **P3** obtained more apparent signals of *sc*-SWNTs (in a range of about 800 to 1600 nm) than **P2** and **P1**. Several crucial parameters were defined to calculate the efficiency of wrapping *sc*-SWNTs. Selectivity (ϕ) means the purity of *sc*-SWNTs, defined as the integral areas of *S*_22_ peak absorbance divided by the sum of the integral of *S*_22_ peak absorbance and the baseline absorbance: ϕ = *A*_S22_/(*A*_S22_ + *A*_baseline_),^38^ where *A*_S22_ is the integral of the S_22_ peak and *A*_baseline_ is the integral of the baseline; yield means the number of *sc*-SWNTs (targeted SWNTs). The yield was calculated by using Beer’s law to evaluate the UV–vis–NIR absorbance: λ = ε *bc*, where ε is the absorption coefficient of *sc*-SWNTs in toluene, *b* means the optical path length, and *c* implies the concentration of the tested solution. After *c* was obtained, the yield could be determined: Yield = (*C*_*sc*-SWNTs_ × *V*)/(2*W*_SWNTs_/3), where *C*_*sc*-SWNTs_ means the concentration of *sc*-SWNTs in the sorting solution, *V* implies the volume of the sorting solvent, and *W*_SWNTs_ means the total weight of SWNTs weighted for the sorting process. The sorting parameters are summarized in Table S3 (Supporting Information). The absorbances of NDI-based CPs were deconvoluted and subtracted from the absorption spectra of CP/*sc*-SWNT solutions. The ϕ values of **P1**, **P2**, and **P3** are 0.33, 0.43, and 0.59, respectively (Figure S19, Supporting Information). From the literature, a high ϕ value means higher purities of *sc*-SWNTs. With a ϕ value >0.33–0.40, the corresponding purity is higher than 99%.^39,40^ Thus, the purity of *sc*-SWNTs sorted by **P1** is approximately 99%, and **P2** and **P3** have purities >99%. The calculated yields are 34.0%, 2.3%, and 23.3%, respectively. As can be seen, **P2** and **P3** have higher purities than **P1**; **P2** has a poor yield due to its poor solubility in toluene, and **P3** has the highest selectivity and sufficient yield for device fabrication.

Furthermore, to further evaluate the properties of *sc-*SWNTs, Raman spectroscopy with 633 nm laser excitation was used to measure the drop-cast film from the sorting solutions. Figure 2b,c displays the Raman spectra’s high-wavenumber and low-wavenumber regions, respectively. As can be seen, the G-band shows the SWNT’s features, composed of two main parts in Figure 2b. The G^+^ band, about 1590 cm^–1^, represents carbon atoms vibrating along the SWNT axis, while the G^–^ band, about 1570 cm^–1^, represents carbon atoms vibrating along the circumferential axis of the SWNT. The D-band refers to a specific characteristic associated with structural defects and impurities in SWNTs. Hence, the G- and D-band ratios can be used to evaluate the structural integrity and purity of SWNT. Due to this, the G/D ratios of reported polymers/*sc*-SWNTs are 9, 15, and 13, meaning that as the side chain modifications are conducted, the defect ratio decreases relatively. Jorio et al. reported the series of Raman spectroscopy of SWNTs, including raw SWNTs and *sc-*SWNTs.^41^ It is worth noting that the peak in the D-band of **P3** is sharper, in other words, narrower than that of **P1**. This disparity indicates that, after the wrapping process, *sc*-SWNTs wrapped by **P3** have more uncomplicated defects than those of **P1**. The broader peak of the D-band might indicate more defects, leading to impurities (such as amorphous carbon) or damage in the structure of *sc*-SWNTs. The G^+^/G^–^ ratio of reported polymers is about 1.95, 1.93, and 5.30, respectively, indicating **P3** performs better in the ability to bundle longer and less-defect *sc*-SWNTs. Su et al. report the Raman spectrum of raw SWNTs containing numerous defects and metallic components. The corresponding G^+^/G^–^ and G/D ratios are approximately 0.94 and 9.2.^42^ The significantly higher ratios achieved by **P3** indicate the efficacy of the conjugated side chain in improving the sorting efficiency of *sc*-SWNTs. In addition, in the low-wavenumber region (Figure 2c), the radial-breathing mode (RBM) is about 160 to 210 cm^–1^, referring to a specific vibration mode, typically attributed to characterizing the diameter distribution of SWNTs. For medium-diameter SWNTs, the diameter can be calculated by *w*_RBM_ = *A*/d*t* + *B*, where *w*_RBM_ is the width of PD-SWNTs, *A* = 234 cm^–1^, and *B* = 10 cm^–1.^^43^ By combining the above equation and data plot, the diameter of PD-SWNTs could be approximately 1.27 nm. The carrier scattering processes are reduced for *sc-*SWNTs in the diameter range of 1.5–2.0 nm because the phonon energy is comparable to the room-temperature thermal energy.^25^ Therefore, the developed CP/*sc-*SWNTs have potential in FET device applications, which will be further investigated in a subsequent section.

With regard to the existence of CPs on *sc-*SWNTs, Fourier–transform infrared (FT–IR) spectroscopy was applied with the drop-cast film mentioned in the last section. In the spectra displayed in Figure 2d, several characteristic peaks were observed: the peaks at 1100 to 1180 cm^–1^ represent the stretching of C–N in imide groups; the peaks at 1730 cm^–1^ represent the stretching of C=O in tertiary amide groups and ester groups; the peaks at 1650 to 2000 cm^–1^ represent the bending of C–H in aromatic compounds; and the peaks at 2800 to 3000 cm^–1^ represent the stretching of C–H in side-chain alkanes. Figure S20 shows the FT–IR spectra of the raw SWNT and the pure polymers of **P1**, **P2**, and **P3**. Notably, compared to the polymer powder, the peaks at 3000 to 3500 cm^–1^ refer to the occurrence of hydrogen bonding caused by the water accidentally mixing in the film during the device fabrication process. The peaks above provide insights into the compositions and structures of CPs in the composites.

### Theoretical Calculations of the Polymer/*sc*-SWNTs

**Figure 3:**
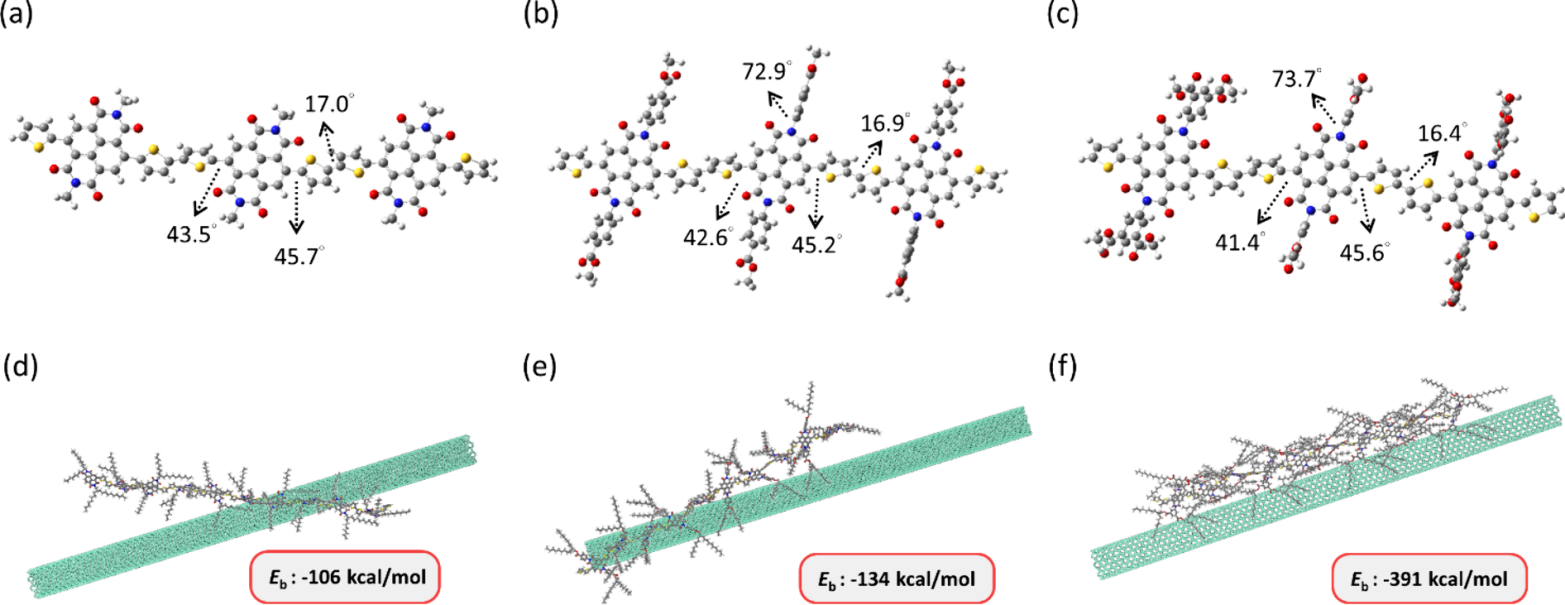
(a–c) Optimized molecular structures of CPs simulated from DFT calculations and (d–f) MD simulated conformation and binding energy of CP/*sc*-SWNT for (a, d) **P1**, (b, e) **P2**, and (c, f) **P3**.

DFT and MD simulations were conducted to further understand how the coplanarity of CPs after side chain modification with different conjugated side groups would impact the whole structure and sorting processes. The DFT calculation was used to optimize the geometry and conformation of the CPs studied, providing a thorough analysis of bond length, bond angles, dihedral angles, etc. In particular, DFT could optimize the dihedral conformers of the CP backbones, elucidating the intrinsic coplanarity of CPs. To simplify the simulations, three NDI and two 2T monomers were used for the calculation and methyl groups were used for replacing long alkyl side chains. As can be seen in Figure 3a–c, P3 has the smallest dihedral angle of 41.4° between the NDI acceptor and the 2T donor units, while **P1** and **P2** have slightly larger angles of 43.5° and 42.6°, respectively. The dihedral angles in the donor unit are similar at 17.0°, 16.9°, and 16.4° for **P1**, **P2**, and **P3**, respectively. This indicates that the increase in alkyl side chains can undoubtedly have an impact on the coplanarity on the backbone with the same donor–acceptor combination. With regard to the side chains, the phenyl groups in **P2** and **P3** rotate to 72.9° and 73.7° in conjunction with the NDI moiety due to their steric hindrances. This outcome reveals that the aggregation behaviors are related to the steric hindrances of conjugated side chains and backbone coplanarity. **P3** with side-chain branching leads to a more rotated side group and coplanar backbone than **P1** and **P2**, thereby giving rise to its low aggregation fraction; also, it implies that a larger plane for covering the SWNTs and more branched side chains for bundling SWNTs are beneficial for enhancing the purity of *sc-*SWNTs.

The MD simulation can simulate the complex interaction within CPs/*sc*-SWNTs and more realistically analyze the absorption energies. Figure 3d–f shows the polymer conformation of a CP series of separate donor–acceptor combinations with ten repeat monomers interacting with a 19.6 nm long (9, 9) armchair SWNT using MD simulations. From the simulation snapshots, we can observe how the CPs attach to the SWNTs via π–π interactions. **P2** and **P3** manifest attachment abilities higher than those of **P1**. Particularly, we observed that **P1** adapts more likely to the P configuration, where the polymer backbone is perpendicular to the SWNT’s surface; in comparison, **P3** is becoming more like the T configuration, as the polymer backbone is parallel to the SWNT’s surface. This concept was first proposed by Gomulya et al.^25^ T configuration can give side chains sufficient space for bundling around the whole SWNT. As the size of the side chains increases, the binding energy (*E*_b_) of CPs/SWNTs becomes more negative at −106, −134, and −391 kcal mol^–1^ for **P1**, **P2**, and **P3**, respectively. The more negative *E*_b_ indicates a better interaction between CP and SWNT. These results suggest that the rise in coplanarities of backbones and side-chain size helps to wrap *sc*-SWNTs;^44^ furthermore, the conjugated side groups and the side-chain branching could reduce aggregation ability and disperse well in the solvent for the sorting process. The above simulations can explain why the ϕ value of **P2** and **P3** improve significantly compared to **P1** and present their superior selectivity. These results are also compatible with DFT calculations.

### Morphological Characterization of the Polymer/*sc*-SWNTs Films

**Figure 4:**
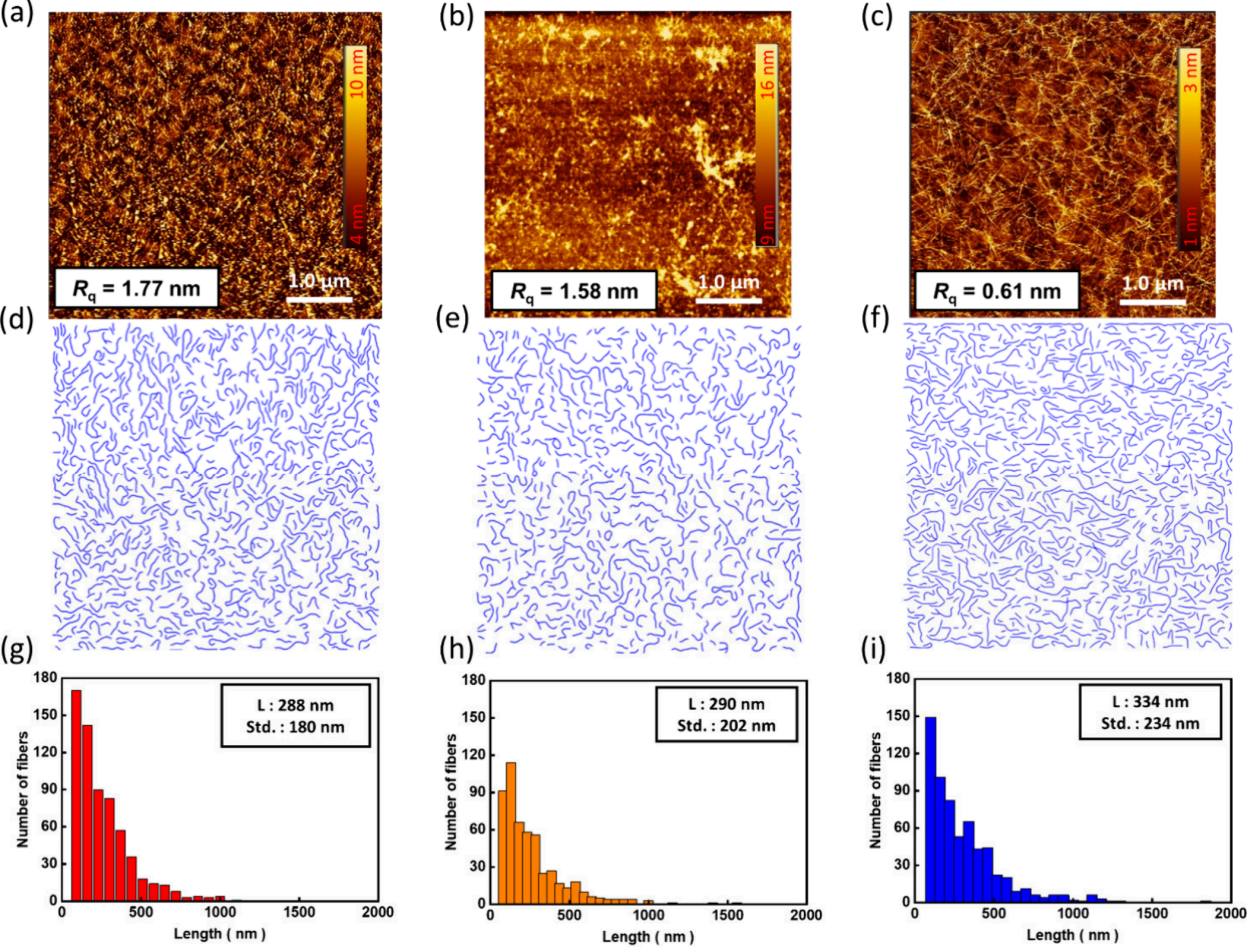
(a–c) AFM topographies, (d–f) nanotube morphology mapping of the AFM image, and (g–i) the statistical length distribution, indicating the average SWNT length and standard deviations of CP/*sc*-SWNT comprising (a, d, g) **P1**, (b, e, h) **P2**, and (c, f, i) **P3**.

After the molecular interactions were investigated, the morphology of polymers/*sc*-SWNTs films is further characterized. With the CP/*sc*-SWNTs solutions, the materials were densely grown onto a hydrophilic substrate, dextran, by sinking the wafer with the sorting solvent. Next, poly(methyl methacrylate) (PMMA) was stuck to the wafer for transferring the CP/*sc*-SWNTs films to a silicon wafer with bilayered thin films of SiO_2_ and cross-linked poly(styrene–butadiene–styrene) (SBS) rubber.^45^ More detailed experimental steps are shown in the Experimental Section. The AFM technique was used, and the topographies are presented in Figure 4a–c. As can be seen, the film of **P1**/*sc*-SWNTs shows several nanotubes lying on the substrate, and some black dots refer to the aggregation of **P1** (Figure 4a). Figure 4b displays the film of **P2**/sc-SWNTs, where barely a few nanotubes can be seen. This is due to the poor solubility of the polymer in toluene, leading to the lowest yield of all; the film of **P3**/sc-SWNTs is shown in Figure 4c, and many nanotubes can be observed clearly in the figure, showing the same outcome compatible with simulations. Then, the relative roughness (*R*_g_) of **P1**, **P2**, and **P3** becomes smoother for 1.77, 1.58, and 0.61 nm, respectively, representing the nanotubes growing more uniformly on the substrate. To realize the morphology of grown CP/*sc*-SWNTs, the mapping technique extracted from the GTFiber program developed by Persson et al. was applied to display the details of nanotubes in Figure 4d–f,^46^ and the corresponding statics of nanotube lengths are shown in Figure 4g–i. The screening parameters of topographic mapping are presented in Figure S21 (Supporting Information). Compared to **P1** and **P2**, **P3** shows more nanotubes, indicating that the wrapping ability of **P3** is better. From the length statics, the film of **P3**/*sc*-SWNTs shows longer nanotubes than that of **P1**/*sc*-SWNTs. Through the side-chain modification and biaxial extension of the backbone, **P3** owns the specialties of longer *sc*-SWNTs with better percolation on the surface. This result strongly relates to the binding energy and coplanarity of CPs, providing better wrapping ability of the *sc*-SWNTs and is compatible with the trend of the G^+^/G^–^ ratio.^44^ The extension of phenyl groups provides a better interaction covering the circumferential axis; thus, the backbone can extend for bundling with *sc*-SWNTs.

**Figure 5:**
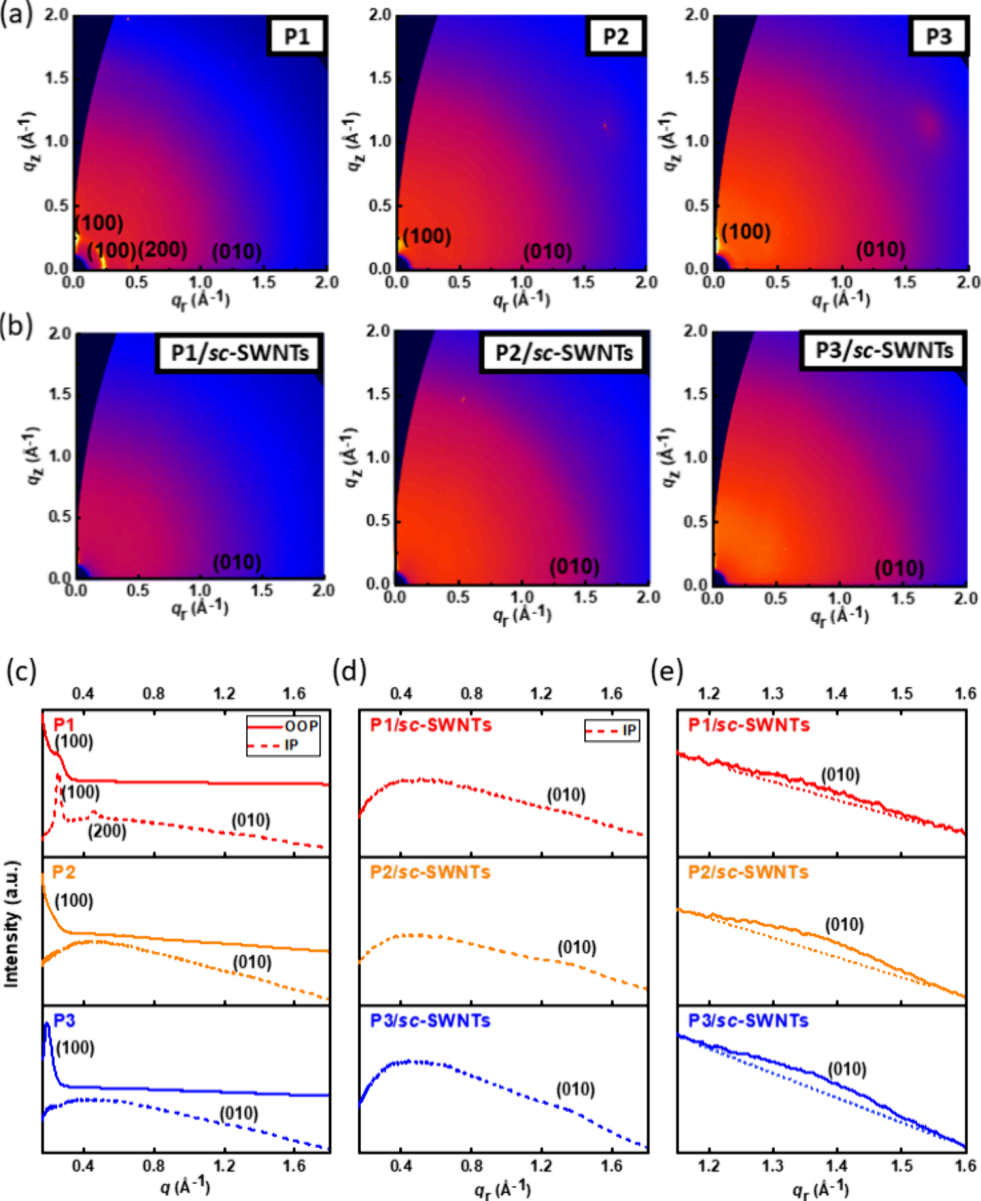
(a, b) 2D GIXD patterns and (c–e) the extracted 1D GIXD line-cutting profiles of (a, c) the spin-coated CP films and (b, d, e) the CP/*sc*-SWNT films. Note that the 1D GIXD profiles were integrated along the OOP/IP directions for the pristine polymer films and the IP direction for the CP/*sc*-SWNT films, and the dotted line inside (e) indicates the baseline of π–π stacking diffraction spanning the range of 1.2 to 1.5 Å^–1^.

GIXD techniques were applied to gain insight into the crystallographic parameters, molecular packing, and orientation of the CP and CP/*sc*-SWNT films. Figure 5a shows the 2D-GIXD patterns of each polymer film; Figure 5b shows the measured 2D-GIXD patterns of CPs/*sc*-SWNTs films, and their corresponding out-of-plane (OOP) and in-plane (IP) 1D-scanning profiles are displayed in Figures 5c,d and S22, respectively. The relevant crystallographic parameters are summarized in Table S3 (Supporting Information) for pristine CPs and in Table S4 (Supporting Information) for CP/*sc*-SWNTs. From Figure 5a,c, along the *q*_*z*_- and *q*_r_-axis in the profiles, **P1** has more obvious (n00) diffraction peaks than **P2** and **P3**. This indicates that the crystallinities are reduced via conjugated side-chain modifications and the face-on orientations are inhibited. The values of lamellar stacking distance (*d*_100_) of each CP are 25.1, 31.9, and 34.3 Å for **P1**, **P2**, and **P3**, respectively. This reveals that introducing conjugated side chains would influence the alignment of the polymers. After the sorting processes, the lamellar stacking peaks along the *q*_*z*_-axis (out-of-plane direction, OOP) are barely seen, but the π–π stacking peaks along the *q*_r_-axis in the in-plane profile (IP, Figure 5d) increase. This result shows that, after bundling *sc*-SWNTs, polymers have weaker lamellar stacking in comparison to pure polymer films. This might be attributed to the polymer chains arranged more closely, forming an alignment parallel to the surface of the nanotubes. In more detail, these profiles show the morphology of CPs/*sc*-SWNTs existing in only π–π stacking along the radial orientation between CPs, owing to the side chains bundling with *sc*-SWNTs. In contrast, lamellar stacking around SWNTs or between CPs does not appear. According to the calculation of IP (010) diffractions in Figure 5d, the π–π stacking distances (*d*_010_) are 4.65, 4.61, and 4.59 Å for **P1**, **P2**, and **P3**, respectively. The CPs with conjugated side chains show narrower π–π stacking than **P1**. This result coincides with the simulated conformations, indicating that **P3** exhibited better backbone coplanarity. In addition, a good π–π stacking between CPs can warrant a compact packing of CP/*sc*-SWNTs in the solid state because of the low steric hindrance between CPs, which is conducive to charge transport in FET devices. Next, the values of paracrystalline disorder (*g*_010_) are 16.9%, 17.5%, and 17.6%, respectively (Table S4, Supporting Information), indicating a similar mechanism for CPs wrapping around SWNTs.^47^ These results benefit the interaction of CPs/*sc*-SWNTs and are favorable for FET device applications.^48^

### FET Device Characterization of Polymer/*sc*-SWNTs

**Figure 6:**
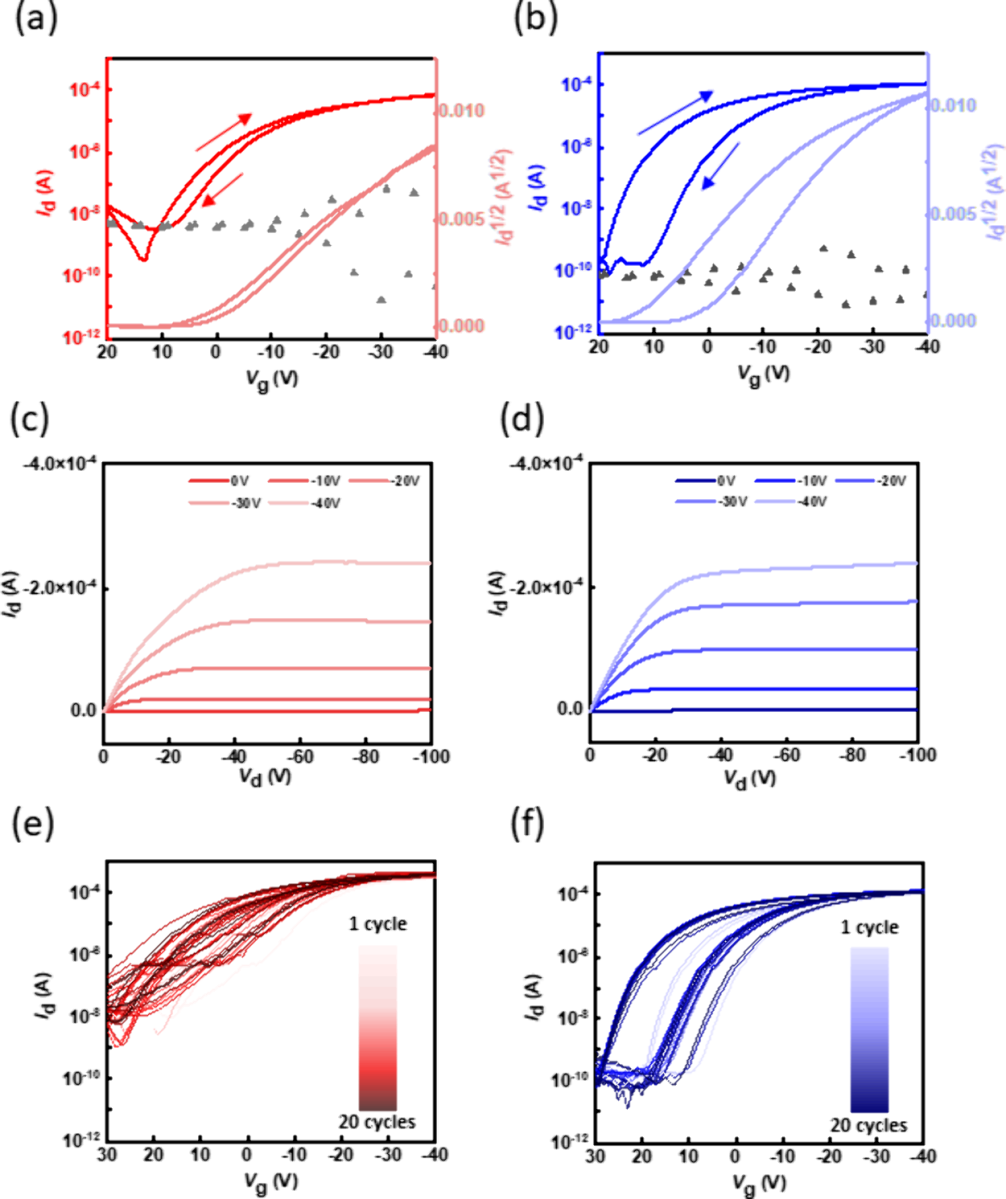
(a, b) Transfer characteristic curves, (c, d) output characteristic curves, and (e, f) endurance tests by applying 20 consecutive transfer sweeps of the devices comprising (a, c, e) **P1**/*sc*-SWNT and (b, d, f) **P3**/*sc*-SWNT. Note that the gray scattered dots represent the gate current measured, and the transfer curve was forwardly swept from 20 to −40 V at *V*_d_ = −10 V for p-type operation.

After realizing the relationships and morphologies inside CPs/*sc*-SWNTs, the FET device performance of CP/*sc*-SWNTs was finally characterized. With the CP/*sc*-SWNTs films transferred on a bilayered thin film of SiO_2_ and cross-linked poly(styrene–butadiene–styrene) (SBS) rubber, the bilayered thin films can serve as the gate dielectrics in FET device operations. Then, the device was thermally deposited with top-contact gold electrodes to form a FET device. The bottom-gate/top-contact (BG/TC) device architecture is illustrated in Figure 1b. The fabrication procedure of the device is detailed in the Experimental Section. At the same time, the yield of **P2** is seriously affected by the solubility, leading to poor ability to fabricate an FET device. Figure 6a,b presents the transfer curves of **P1** and **P3**, showing typical p-type characteristic curves managed by the channel of *sc*-SWNTs. The corresponding hole mobility (μ_h_), threshold voltage (*V*_th_), and on–off current ratio (*I*_on_/*I*_off_) are summarized in Table S5 (Supporting Information). The device parameters were averaged among five devices per batch and from two batches. Transfer characteristics were recorded by sweeping the gate voltage (*V*_g_) from 20 to −40 V, as can be seen in Figures 6a,b and S23; the maximum drain current can reach as high as 10^–4^–10^–3^ A. Accordingly, the μ_h_ values of **P1** are 0.48 ± 0.03 cm^2^ V^–1^ s^–1^ at *V*_d_ = −10 V and 2.21 ± 0.12 cm^2^ V^–1^ s^–1^ at *V*_d_ = −100 V; those for **P3** are 0.99 ± 0.08 cm^2^ V^–1^ s^–1^ at *V*_d_ = −10 V and 4.72 ± 0.58 cm^2^ V^–1^ s^–1^ at *V*_d_ = −100 V, indicating that, through side-chain modification, the mobility of **P3**/*sc*-SWNTs as the channel layer gets significantly increased. This result is compatible with the findings discussed in the morphology section, attributed to higher purity, stronger binding energy, and longer *sc*-SWNTs. Through calculation, the corresponding maximum transconductance (*g*_m_) values of **P1** and **P3** are 4 and 7 nS/μm. This indicates that, compared to **P1**, **P3** has a smaller change in *V*_g_, leading to a significant change in *I*_d_, making the device more efficient in amplifying signals.^49^ In addition, the relatively low *g*_m_ can be attributed to the low capacitance of these devices fabricated based on 300 nm-thick SiO_2_ wafers. With regard to the device hysteresis and *V*_th_ values, Dallaire et al. reported that using an octyltrichlorosilane (OTS) modified device process and appropriate wavelength of light exposure to the device would lower the *V*_th_ for about 4 V and the dark currents, reducing the activation energy required for activating the device.^50^ The OTS dielectric layer would provide relatively low interfacial charge traps due to the hydrophobic characteristics, leading to better electric properties. While using dextran and SBS for fabricating devices in the film-transfer process in this work, we could grow more CPs/*sc*-SWNTs for our device. In this work, the AFM topographies show significantly the whole scene of *sc*-SWNTs. Compared to the reported system,^31^ the μ_h_ values of the device with pure *sc*-SWNTs (5.3 cm^2^ V^–1^ s^–1^) are about five times higher than the devices in this study. This disparity shows the different charge affinities between *sc*-SWNTs and these n-type CPs. While **P3** has a more considerable hysteresis of about 8 V, it might be the combinatory result of adsorbed water and oxygen present at the interface between the channel and dielectric layers caused by the film-transfer process in fabricating the device.^51−53^ In addition, Srimani et al. applied an advanced *sc*-SWNT purification approach by adding silica gel particles after the first centrifugation, which can further lower the dark current of the device.^54^ Notably, **P3** has a better *I*_on_/*I*_off_ ratio because the dark current of **P3** is lower than that of **P1** (Figure 6a,b). The dark current is dependent on charge traps and *sc*-SWNT packing densities.^50^**P1** has higher SWNT densities (higher yield) and trap densities than **P3**, leading to a relatively higher dark current caused by the higher *m*-SWNTs and amorphous carbon contents, as seen in the broad background in Figure S19a. The quantized trap density is further discussed in the subsequent section.

As for the device comprising **P2**/*sc*-SWNTs, Figure S24 (Supporting Information) displays the corresponding transfer and output curve of the device. The low yield of **P2**/*sc*-SWNT gives rise to its poor device performance. Figure 6c,d displays **P1** and **P3**’s output curves, showing that drain currents can be scaled when *V*_g_ is given. With the same *V*_g_, **P3**/*sc*-SWNT turns to on-state faster at a smaller *V*_d_ than **P1**/*sc*-SWNT. This electrical property enables the device to switch faster, as it can quickly respond to the saturation state with lower voltages. In addition, the saturated on-state currents of **P3**/*sc*-SWNT at different *V*_g_ are also higher than those of **P1**/*sc*-SWNT. Although the general mobility of the device with commercial SWNTs is still about an order higher than that in this study, the sorting processes with specific CPs can greatly help wrap the consistent length of *sc*-SWNTs without impurities and different chiralities taken from commercial SWNTs,^55,56^ and the scalability of CP/*sc*-SWNT has potential.

Regarding pure CPs for the channel layers (Figure S25, Supporting Information), the typical n-type characteristic curves and the electron mobility (μ_e_) are low enough to be neglected in conjunction with *sc*-SWNT. The device data are summarized in Table S6 (Supporting Information). Nevertheless, to preclude the contribution of electron transport, n-type operations with *V*_g_ swept from −20 to 40 V were conducted again to check whether the CPs formed a conductive channel. The transfer characteristics of **P1**/*sc*-SWNTs and **P3**/*sc*-SWNTs are demonstrated in Figure S26 (Supporting Information). As seen in the profiles, the drain currents in the positive *V*_g_ range are 1,000 times lower than those in the negative *V*_g_ range. Therefore, μ_e_ is much smaller than μ_h_, indicating that the n-type channel does not constitute the conduction.

With regard to operational stability, Figure 6e,f displays consecutive sweeping profiles. Although the mobilities were the same, the off current of **P1**/*sc*-SWNTs increased after 20 cycles, and |*V*_th_| became higher. It reveals its instability in electrical endurances. However, **P3**/*sc*-SWNTs maintained almost the same trend in transfer characteristics as the first cycle after sweeping 20 times, indicating better endurance and stability. For quantifying this instability, we further applied the following equation: *N*_tr_ = *C*_i_·[*qS*/*k*_B_*T*·ln(10) – 1]/*q*, where *q* is the elementary charge, *S* is the subthreshold swing (V/decade), *N*_tr_ is the maximum interfacial trap density estimated from *S*, *k*_B_ is Boltzmann’s constant, and *C*_i_ is the capacitance of the bilayered gate dielectrics (8.61 nF cm^–2^). The calculated results are summarized in Table S5 (Supporting Information). Compared to **P3**, **P1** has a higher *N*_tr_, indicating a more significant number of interfacial hole traps, which is relatively detrimental to the device operation.^57^ Next, a bias-stress test was conducted to observe their long-term stability. The time-dependent stability of the drain current was recorded under constant bias stress of *V*_g_ = −10 V and *V*_d_ = −1 V for over 9000 s (Figure S27, Supporting Information). The corresponding transfer curves at *V*_d_ = −1 V are displayed in Figure S28 (Supporting Information) to confirm the current levels observed in the bias stress test. As seen in the profiles, the drain current of **P1** is not as stable as that of **P3**, although **P3** still has an increasing current.^58^ These results can be attributed to the poor aggregation of **P3** and the better ability of bundle *sc*-SWNTs to fill up the interface between them. Although the gating effect on the *sc*-SWNTs channel is not negligible, it can be further improved by testing the device in a nitrogen atmosphere.

## Conclusion

In conclusion, side-chain modified CPs with biaxially extended conjugations were synthesized and applied for wrapping *sc*-SWNTs. With modifications, the increasing steric hindrances sufficiently disperse CPs well in the toluene. The alkyl-phenyl groups as side chains not only enlarge the side chain pattern but also rotate the side chain moieties proximal to the backbones, so the CP’s aggregation behavior decreases. The aggregation behaviors are related to the steric hindrances of conjugated side chains and the backbone coplanarity. The conjugated side groups and the side-chain branching could reduce aggregation ability and disperse well in the solvent for the sorting process. The phenyl groups provide sufficient spaces for the alkyl chains, making them available to bundle *sc*-SWNTs. As the size of the side chains increases, the interaction between CP/*sc*-SWNTs is enhanced. The simulated result is compatible with the experimental findings. The high performance of **P3**/SWNTs is attributed to the higher purity, stronger binding energy, longer *sc*-SWNTs, and narrower π–π spacing between the CPs. Accordingly, the device composed of **P3**/*sc*-SWNTs exhibited better device performance with higher μ_h_ of 4.72 cm^2^ V^–1^ s^–1^ than that of 2.21 cm^2^ V^–1^ s^–1^ for **P1**/*sc*-SWNTs. In addition, **P3**/*sc*-SWNTs showed better sweeping endurance stability than did **P1**/*sc*-SWNTs. In comparison, the performance of **P2**/*sc*-SWNTs is confined due to their poor solubility. In summary, side-chain modifications with biaxially extended conjugation are conducive to improving the sorting efficiency of NDI-based CPs with respect to *sc*-SWNTs. The purer *sc*-SWNTs give rise to advanced FET device performance. The underlying structure–performance relationship of biaxially extended CPs deserves further investigation.

## Experimental Section

### Materials

Naphthalene-1,4,5,8-tetracarboxylic dianhydride (>97%), dibromoisocyanuric acid (>97%), 4-aminobenzoic acid (>99%), 5-aminoisophthalic acid (>94%), acetic acid glacial (>99.7%), 2-octyl-1-dodecanol (ODOH, >97%), 4-(dimethylamino)pyridine (DMAP, >99%), N1-((ethylimino)methylene)-N3,N3-dimethylpropane-1,3-diamine hydrochloride (EDC-HCl, >95%), tris(dibenzylideneacetone)dipalladium(0) (Pd_2_(dba)_3_, >98%), 5,5′-bis(trimethylstannyl)-2,2′-bithiophene (2T, >97%), tri(*o*-tolyl)phosphine (P(*o*-tolyl)_3_, >98%), anhydrous chlorobenzene (CB, >99.8%), 2-bromothiophene (>98%), 2-(tributylstannyl)thiophene (>97%), dextran, poly(methyl methacrylate) (PMMA), pentaerythritol tetrakis(3-mecraptopropionate) (>95%), phenylbis(2,4,6-trimethylbenzoyl) (>97%), and polystyrene-*block*-polybutadiene (30% styrene, SBS) were purchased from Sigma-Aldrich, Tokyo Chemical Industry Co., Thermo Fisher Scientific, Acro Organics B.V.B.A., DUKSAN, Alfa Aesar, Ultra Fine Chemical Technology Corp., and Luminescence Technology Corp. Plasma discharge-SWNTs (PD-SWNTs) were ordered from Yuang Hong Inc. and NanoIntegris Inc. The above chemicals were used as received without further purification. 2,6-Dibromonaphthalene-1,4,5,8-tetracarboxylic dianhydride (Br-NDA-Br) and 4,9-dibromo-2,7-bis(2-octyldodecyl)benzo[*lmn*][3,8]-phenanthroline-1,3,6,8(2*H,*7*H*)-tetraone (Br-NDI-Br) were synthesized following the reported method.^59^

### Synthesis of 4,4′-(4,9-Dibromo-1,3,6,8-tetraoxo-1,3,6,8-tetrahydrobenzo[*lmn*][3,8]phenanthroline-2,7-diyl)dibenzoic Acid (**M1**)

Br–NDA–Br (320 mg, 0.75 mmol), 4-aminobenzoic acid (309 mg, 2.25 mmol), and glacial acetic acid (5 mL) were placed in a 50 mL flask. The solution was then stirred and refluxed under N_2_ at 100 °C overnight. After the reaction, the solution was poured into water and dried; subsequently, it was dissolved in methanol and precipitated in dichloromethane to afford **M1** as a dark red solid (345 mg, 69%). ^1^H NMR (500 MHz, DMSO-*d*_6_, δ ppm, 25 °C, Figure S2): 8.78 (s, 2H), 8.13 (t, *J* = 16 Hz, 4H), 7.6 (d, *J* = 8 Hz, 4H). ^13^C NMR (100 MHz, DMSO-*d*_6_, δ ppm, 25 °C, Figure S3): 119.45, 122.24, 124.83, 125.89, 129.99, 130.91, 134.50, 136.07, 138.52, 142.79, 160.86, 164.54, 166.83. Anal. Calcd for C_28_H_12_Br_2_N_2_O_8_ (%): C, 50.6; H, 1.8; N, 4.2. Found (%): C, 48.8; H, 1.9; N, 3.9.

### Synthesis of 5,5′-(4,9-Dibromo-1,3,6,8-tetraoxo-1,3,6,8-tetrahydrobenzo[*lmn*][3,8]phenanthroline-2,7-diyl)diisophthalic Acid (**M2**)

Br–NDA–Br (320 mg, 0.75 mmol), 5-aminoisophthalic acid (406 mg, 2.25 mmol), and acetic acid glacial (5 mL) were placed in a 50 mL flask. The solution was then stirred and refluxed under N_2_ at 100 °C overnight. After the reaction, the solution was poured into water and dried; subsequently, it was dissolved in methanol and precipitated in dichloromethane to afford **M2** as a dark red solid (542 mg, 96%). ^1^H NMR (500 MHz, DMSO-*d*_6_, δ ppm, 25 °C, Figure S4): 8.78 (s, 2H), 8.59 (t, *J* = 3.5 Hz, 2H), 8.32 (d, *J* = 1.5 Hz, 4H). ^13^C NMR (100 MHz, DMSO-*d*_6_, δ ppm, 25 °C, Figure S5): 124.29, 126.24, 128.05, 131.62, 132.29, 134.22, 136.38, 139.86, 161.06, 165.93, 168.77, 169.55. Anal. Calcd for C_30_H_12_Br_2_N_2_O_12_ (%): C, 47.9; H, 1.6; N, 3.7. Found (%): C, 48.3; H, 3.0; N, 5.0.

### Synthesis of Bis(2-octyldodecyl)4,4′-(4,9-dibromo-1,3,6,8-tetraoxo-1,3,6,8-tetrahydrobenzo[*lmn*][3,8]phenanthroline-2,7-diyl)dibenzoate (**M3**)

ODOH (664 mg, 2.2 mmol), DCM (4.2 mL), DMF(0.8 mL), DMAP (61 mg, 0.5 mmol), EDC-HCl (480 mg, 2.5 mmol), and **M1** (664 mg, 1 mmol) were placed in a 50 mL flask. The solution was stirred under N_2_ at 50 °C overnight. The solution was rotary-evaporated and extracted with dichloromethane and water for a slurry-like crude product. Then, the mixture was rinsed with methanol to afford **M3** as a light red solid (210 mg, 17%). ^1^H NMR (500 MHz, CDCl_3_, δ ppm, 25 °C, Figure S6): 9.08 (s, 2H), 8.26 (d, *J* = 8.5 Hz, 4H), 7.42 (d, *J* = 8.5 Hz, 4H), 4.29 (d, *J* = 5 Hz, 4H), 1.81 (q, *J* = 23.5, 12 Hz, 2H), 1.55 (s, 4H),1.34–1.22 (br, 66H), 0.91–0.85 (br, 6H). ^13^C NMR (100 MHz, CDCl_3_, δ ppm, 25 °C, Figure S7): 14.10, 22.67, 26.79, 29.34, 29.57, 29.66, 29.98, 31.90, 124.47, 125.64, 128.27, 128.66, 129.25, 130.94, 131.65, 138.18, 139.64, 160.61, 160.68, 165.71. Anal. Calcd for C_68_H_92_Br_2_N_2_O_8_ (%): C, 62.2; H, 7.0; N, 2.1. Found (%): C, 66.3; H, 7.0; N, 3.5.

### Synthesis of Tetrakis(2-octyldodecyl)5,5′-(4,9-dibromo-1,3,6,8-tetraoxo-1,3,6,8-tetrahydrobenzo[*lmn*][3,8]phenanthroline-2,7-diyl)diisophthalate (**M4**)

ODOH (1090.3 mg, 3.65 mmol), DCM (4.5 mL), DMF(1.0 mL), DMAP (61 mg, 0.5 mmol), EDC-HCl (796 mg, 4.15 mmol), and **M2** (626.5 mg, 0.83 mmol) were placed in a 50 mL flask. Then, the solution was stirred and refluxed under N_2_ at 50 °C overnight. The solution was rotary-evaporated and extracted with dichloromethane and water to afford a slurry-like mixture. The mixture was washed with methanol to afford **M4** as a light red solid (225 mg, 14.5%). ^1^H NMR (500 MHz, CDCl_3_, δ ppm, 25 °C, Figure S8): 8.90 (s, 2H), 8.82 (t, *J* = 3.0 Hz, 2H), 8.19 (d, *J* = 2.0 Hz, 4H), 4.29 (d, *J* = 5.5 Hz, 8H), 1.80 (q, *J* = 24.0, 12.0 Hz, 8H), 1.55 (s, 8H), 1.33–1.19 (br, 128H), 0.90–0.83 (br, 12H). ^13^C NMR (100 MHz, CDCl_3_, δ ppm, 25 °C, Figure S9):14.09, 22.67, 26.89, 29.33, 30.93, 31.90, 37.43, 40.54, 65.77, 68.49, 122.67, 126.25, 127.67, 131.33, 133.90, 134.67, 136.59, 141.16, 160.32, 160.71, 164.80. Anal. Calcd for C_110_H_172_Br_2_N_2_O_12_ (%): C, 69.0; H, 9.0; N, 1.5. Found (%): C, 70.3; H, 8.3; N, 3.3.

### General Procedure for Migita–Kosugi–Stille Coupling Polymerization

The synthetic route for CPs is shown in Scheme 1. For a typical synthesis of **P1**, Br–NDI–Br (705 mg, 0.64 mmol), 2T (316 mg, 0.64 mmol), and CB (6.4 mL) were placed into a 50 mL two-necked flask. To this solution, N_2_ was bubbled in for 30 min. Pd_2_(dba)_3_ (58.8 mg, 0.064 mmol) and P(*o*-tolyl)_3_ (195.6 mg, 0.64 mmol) were added to the solution, and the solution was stirred and refluxed under N_2_ at 120 °C for 24 h. After the reaction, 2-bromothiophene (19 μL) and 2-(tributylstannyl)thiophene (61 μL) were individually added to end-cap the reaction at 120 °C for 2 h. After the reaction was cooled to room temperature, the crude polymer was precipitated in methanol and suction-filtered for a dark blue solid product. Then, the product was further purified by Soxhlet extraction using acetone and *n*-hexanes and recovered by chloroform (24 h for each). After rotary-evaporating chloroform, the polymer was redissolved in chloroform for saturation and precipitated in methanol again to afford a dark blue solid (550 mg, 78.2%; the ^1^H and ^13^C NMR spectra are shown in Figures S10 and S11).

*P2.***M3** (197 mg, 0.16 mmol), 2T (78.7 mg, 0.16 mmol), Pd_2_(dba)_3_ (8.8 mg, 0.016 mmol), P(*o*-tolyl)_3_ (14.6 mg, 0.05 mmol), CB (8 mL), 2-bromothiophene (4.7 μL), and 2-(tributylstannyl)thiophene (10.2 μL). Dark green solid (100 mg, 55%; the ^1^H and ^13^C NMR spectra are shown in Figures S12 and S13).

*P3.***M4** (200 mg, 0.11 mmol), 2T (52.6 mg, 0.11 mmol), Pd_2_(dba)_3_ (8.6 mg, 0.01 mmol), P(*o*-tolyl)_3_ (9.8 mg, 0.03 mmol), CB (5.2 mL), 2-bromothiophene (3.1 μL), and 2-(tributylstannyl)thiophene (12.3 μL). Dark green solid (130 mg, 67%; the ^1^H and ^13^C NMR spectra are shown in Figures S14 and S15).

### Sorting Procedure of *sc*-SWNTs

The n-type CPs of 5 mg were dissolved in toluene (20 mL), and the solution was fully dissolved using the ultrasonic cleaner DC300H (DELTA Ultrasonic Co., Ltd.). Then, PD-SWNTs (10 mg) were added with a weight ratio of CP/SWNTs = 1:2. The mixture was then sonicated with 40% amplitude for 30 min by using a VCX750 (Sonic & Materials, INC.), and isopropanol was used for an ice bath to maintain −60 °C. Next, the sorting solutions were centrifuged at 12 000 rpm (relative centrifugal force, RCF = 27 300*g*) and 25 °C for 1 h on a FL3012 (FANLINYL). Finally, the sorting solutions with enriched *sc*-SWNTs were extracted without the remaining impurities in the SWNTs. Note that, when sorting SWNTs with **P2**, the solution was sonicated at 20 °C because **P2** would precipitate at low temperature, resulting in a poor yield.

### Fabrication of FET Devices

Through sequential bath sonication, the Si/SiO_2_ wafers with an oxide thickness of 300 nm and size of 1.5 × 1.5 cm were cleaned via toluene, isopropanol, acetone, deionized water, and ethanol. Wafers were dried with N_2_ and cleaned in an oxygen plasma for 5 min. To fabricate the FET with a BG/TC configuration, an aqueous dextran solution (30 mg mL^–1^) was spin-coated on a wafer at 1000 rpm for 60 s. After spin-coating, the wafer was dried at 140 °C for 10 min to remove the remaining water. Then, the wafer was soaked in a diluted CP/*sc*-SWNT sorting solution (mother liquid/fresh toluene = 2:1 vol/vol) for 3 days. After the process, the wafer was rinsed with more than 20 mL of toluene 4 times to remove excess unbound polymer and solution;^60^ then, the wafer was spin-coated with PMMA (40 mg mL^–1^ in toluene) at 1000 rpm for 60 s. SBS, phenylbis(2,4,6-trimethylbenzoyl), and pentaerythritol tetrakis(3-mercaptopropionate) had a weight ratio of 25:1:1 in toluene (solid content: 1.6 wt %). The solution was spin-coated on another piece of silicon wafer with a 300 nm-thick SiO_2_ layer, and then, the film was heated at 120 °C for 10 min and photo-cross-linked to serve as a polymer dielectric. For the film peeling process, the bilayered PMMA/*sc*-SWNT film was stuck with tape and the film was peeled off from the wafer by sinking it into a water bath. For the film-transfer process, the film was transferred to the SBS-coated wafer with a covered drop of water and we waited until the water evaporated. Subsequently, the film was cut off, and a 40 nm-thick Au layer was thermally deposited with a channel length (*L*) and width (*W*) of 100 and 2000 μm, respectively, through a mask to obtain the top-contact electrode.

### Characterization

The chemical structure, thermal, and electrochemical characterization approaches are described in the Supporting Information. UV–vis–NIR absorption spectra were recorded using Jasco V-770 scanning from 200 to 1600 nm. To calculate the degree of aggregation of reported CPs in toluene, the CPs were dissolved in toluene and 1-chloronaphthalene at 0.25 and 0.05 mg mL^–1^, respectively, and the latter could represent the disorder fraction. The aggregation fraction of CPs in toluene could be obtained by comparing the optical absorption of two samples, and the calculation was based on the reported method.^61^ Raman and FT–IR spectra of the CPs/*sc*-SWNTs films drop-cast on the glass substrate were measured with an excitation wavelength of 633 nm by a UniDRON (CL Technology Co., Ltd.) and a Nicolet 6700 (Thermo Scientific) spectrometer. The morphology of the CPs/*sc*-SWNTs films was probed by an AFM100plus (Hitachi), and the film thickness was measured using an Alpha-Step D-300 (KLA). Grazing incidence X-ray diffraction (GIXD) profiles of the CPs and CPs/*sc*-SWNT films were determined on beamlines 13A1 at the National Synchrotron Radiation Research Center (NSRRC), Taiwan, with an X-ray wavelength of 1.027 Å and an incident angle at 0.12°. For the calculation of paracrystalline disorder (*g*), , where Δ_*q*_ is the full width at half the maximum of a diffraction peak and *d*_*hkl*_ is the interplanar separation along the crystallographic direction.^62^ All FET performance was documented using a Keithley 4200-SCS semiconductor parameter analyzer (Keithley Instruments, Inc.). Note that the testing was performed under ambient conditions. The capacitance of gate dielectric was determined as follows: , where *C*_total_ is the areal capacitance of the SiO_2_ and SBS bilayered dielectrics,^63^*C*_SiO2_ is the areal capacitance of SiO_2_, and *C*_SBS_ is the capacitance of SBS. The hole mobility (μ_h_) and threshold voltage (*V*_th_) were calculated following the slope or extrapolation of the square root of drain-to-source current (*I*_ds_^1/2^) versus gate voltage (*V*_g_) in the saturation region of the transfer curves: , where *W* and *L* are the width and length of the channel electrodes. The transconductance (*g*_m_) is a key parameter in FETs. It measures the device’s ability to convert a change in *V*_g_ to a change in *I*_d_, and the relationship is defined as *g*_m_ = d*I*_d_/d*V*_g_. For the saturation region, .

### Simulation

For the DFT calculations, the Avogadro software was applied to minimize the energy of chemical structures of the polymers under the Merck molecular force field 94 static variant (MMF94s) force field.^64^ Then, the structure was applied for the Gaussian09 W program with the Becke, 3 parameters, Lee–Yang–Parr (B3LYP) method, and a 6-31G basis to obtain the optimizing ground state configurations of the chemical structures. Note that the polymer’s structure was represented using three repeating units, and the alkyl side-chain was replaced with a methyl group to simplify the simulation. The MD calculations followed the reported method to evaluate the interaction within CPs/*sc*-SWNTs.^65^ The simulations interacted with the armchair (9,9) SWNTs under vacuum, and the length was set as 19.67 nm (12 repeats). In the software framework of Material Studio, the COMPASSIII force field was used as an adsorption locator tool to simulate the attachment of CPs on the surface of SWNTs.^66^ Note that the CPs with ten repeating units were attached to the surface of SWNTs to simulate their interactions and binding energy by applying one cycle with two million steps.

## Supporting Information Available

Thermal analysis of TGA and DSC profiles for the polymers; NMR spectra, GPC profiles, and electrochemical characteristics of the polymers; FET transfer/output characteristics of the reference devices; 1D GIXD profiles of the CP/*sc*-SWNT along the out-of-plane direction and the relevant crystallographic parameters of the films (PDF)
