# Supplementary material for: High-Performance Semiconducting Carbon Nanotube Transistors Using Naphthalene Diimide-Based Polymers with Biaxially Extended Conjugated Side Chains
Source: ACS Appl Mater Interfaces. 2024 Aug 13;16(34):45275–88. doi: 10.1021/acsami.4c08981 (PMC11367582; doi:10.1021/acsami.4c08981)
Supplement: Supplementary file 1 — am4c08981_si_001.pdf [file am4c08981_si_001.pdf]

Supporting Information

High-Performance Semiconducting Carbon Nanotube Transistors Using Naphthalene Diimide-based Polymers with Biaxially Extended Conjugated Side Chains

Chun-Chi Chen,[a] *Shang-Wen Su,*[a] *Yi-Hsuan Tung,*[a] *Po-Yuan Wang,*[a] *Sheng-Sheng Yu,*[a] *Chi-Cheng Chiu,*[a] *Chien-Chung Shih*[c,*] *and Yan-Cheng Lin,*[a,b,*]

[a] Department of Chemical Engineering, National Cheng Kung University, Tainan 70101, Taiwan

[b] Advanced Research Center for Green Materials Science and Technology, National Taiwan University, Taipei 10617, Taiwan

[c] Department of Chemical Engineering and Materials Engineering, National Yunlin University of Science and Technology, Yunlin 64002, Taiwan

[*]Corresponding author. E-mail:
shihcc@yuntech.edu.tw (C.-C. Shih); ycl@gs.ncku.edu.tw (Y.-C. Lin);

Table S1. Optical, electrochemical, and thermal properties of the polymers.

CPs	λ_{max} (nm) [a]	HOMO (eV) [b]	LUMO (eV) [b]	E_g^{cv} (eV) [c]	E_g^{UV} (eV) [d]	T_d (°C) [e]
**P1**	389, 705	-5.96	-4.09	1.87	1.76	436
**P2**	369, 637	-5.91	-3.77	2.14	1.95	312
**P3**	375, 635	-5.89	-3.73	2.16	1.95	358

[a] UV-Vis absorption maximum position of the CPs dissolved in toluene. [b] Derived from the CV oxidative onset potential determined using Fc/Fc^+ as an internal potential reference. [c] HOMO and LUMO gap determined from the difference between the oxidative and reductive onsets in CV profiles. [d] Energy bandgap determined from the onset wavelength in UV-Vis spectrum. [e] Determined from TGA at 5% weight loss in a nitrogen atmosphere.

Table S2. FET dielectric layer parameters.

Layer	Thickness (nm)	Dielectric constant (ε_r)
**SiO_2**	300	3.9
**SBS**	30	2.4

Table S3. Summary of the sorting parameters of CP/*sc*-SWNT solutions and the crystallographic parameters of the pristine CP films.

CPs	$C_{sc\text{–}SWNTs}$ (g L^{-1})[a]	ϕ [a]	Purity (%)	Yield (%)	d_{100} (Å) [b]	q_{100} (Å^{-1}) [b]
**P1**	0.044	0.325	~99	34.0	25.1	0.25
**P2**	0.003	0.429	>99	2.3	31.9	0.20
**P3**	0.032	0.586	>99	23.3	34.3	0.18

[a] *sc*-SWNT concentration of the as-sorted solutions and ϕ value determined from the UV−Vis absorption spectra in **Figure S19**. [b]*d*-spacing and *q* values are extracted from the 1D GIXD profiles.

Table S4. Crystallographic parameters of the CP/*sc*-SWNT films, including the *d*-spacing, full-width at half-maximum (FWHM), and paracrystalline disorder of the IP(010) diffraction peaks.

CPs/*sc*-SWNTs	d_{010} (Å)	q_{010} (Å^{-1})	FWHM	g_{010} (%)
**P1**	4.65	1.352	0.245	16.9
**P2**	4.61	1.362	0.262	17.5
**P3**	4.59	1.369	0.267	17.6

Table S5. Device performances of FETs comprising the CP/*sc*-SWNTs.

CPs/*sc*-SWNTs	μ_h (cm^{2}V^{-1}s^{-1})^a	V_{th} (V)^b	I_{on}/I_{off}^{c}	S_s (V/dec)^d	N_{tr} (× 10^{12} cm^{-1}eV^{-1})^e
**P1** (V_d = –1 V)	0.058	3.8	10^5	5.8	5.27
**P3** (V_d = –1 V)	0.079	11.1	10^5	4.6	4.18
**P1** (V_d = –10 V)	0.48	4.4	10^5	6.3	5.73
**P3** (V_d = –10 V)	0.99	11.6	10^6	5.6	5.09
**P1** (V_d = –100 V)	2.21	13.7	10^5	15.2	13.8
**P3** (V_d = –100 V)	4.72	24.5	10^3	12.6	11.5

^{a} Average hole mobility of the P-type FET device measured at V_d = –10 or –100 V. ^{b} Threshold voltage. ^{c}On-off current ratio. ^{d} Subthreshold swing. ^{e} Maximum interfacial trap density calculated from the subthreshold slope.

Table S6. Device performances of FETs comprising the CP thin films as the channel.

CPs	μ_e (cm^{2}V^{-1}s^{-1})^a	V_{th} (V)^b	I_{on}/I_{off}^{c}
**P1**	3.5×10^{-2}	37.0	10^3
**P2**	1.8×10^{-3}	14.4	10^5
**P3**	3.4×10^{-5}	27.7	10^3

^{a} Average electron mobility of the N-type FET device measured at V_d = 100 V. ^{b}Threshold voltage. ^{c}On-off current ratio.

Materials Characterizations.

^{1}H and ^{13}C nuclear magnetic resonance (NMR) spectra were recorded on a Bruker AVNEO500 with working frequencies of 500 MHz for ^{1}H and 125 MHz for ^{13}C, respectively, and with *d*-chloroform ($CDCl_3$) and dimethyl sulfoxide-d_6 (DMSO-d_6) as the *d*-solvent. Number-average molecular weights (M_n), weight-average molecular weights (M_w), and PDI of polymers were measured at 40 °C and flowing rate of 0.3 mL min^{-1} in tetrahydrofuran (THF) by gel permeation chromatography (GPC) on a LC-20AT (Shodex GPC LF-604). Thermogravimetry analysis (TGA) measurement was performed from 100 to 750 °C with a heating rate of 10 °C min^{-1} on a Perkin Elmer TGA4000. Differential scanning calorimetry (DSC) was measured under N_2 protection from 30 to 300 °C with a heating rate of 10 °C min^{-1} on a Perkin Elmer DSC6000. The elemental analysis of reported polymers was performed on a UNICUBE (Elementar, Germany). Cyclic voltammetry (CV) analysis was conducted by a CHI 6273E electrochemical analyzer (CH Instrument Inc.), and ITO glass, Pt wire, and Ag/$AgNO_3$ (acetonitrile (*sat.*)) were served in a three-electrode cell system as the working, auxiliary, and reference electrodes, respectively.

Physical properties of the synthesized CPs:

P1: ^{1}H NMR (500 MHz, $CDCl_3$, δ ppm, 25 °C, **Figure S10**): 8.67 (d, J = 155 Hz, 2H), 7.48–7.07 (br, 4H), 4.11 (s, 4H), 1.98 (s, 2H) 1.51–0.78 (br, 76H). M_n (GPC) = 66000, PDI = 1.69. Yield = 78% (550 mg).

P2: ^{1}H NMR (500 MHz, $CDCl_3$, δ ppm, 25 °C, **Figure S12**): 8.89 (s, 2H), 8.22 (d, J = 5 Hz, 4H), 7.41 (d, J = 6 Hz, 4H), 7.32 (s, 2H), 7.22 (s, 2H) 4.20 (d, J = 5.5 Hz, 4H), 1.58 (s, 2H), 1.44–0.79 (br, 76H). M_n (GPC) = 5,200, PDI = 1.23 (bimodal molecular weight distribution due to the polymer aggregates). Anal. Calcd for $C_{76}H_{98}N_2O_8S_2$ (%): C, 74.1; H, 8.9; N, 2.3; S, 5.2. Found (%): C, 67.2; H, 7.4; N, 2.14; S, 3.6. Yield = 55% (100 mg).

P3: ^{1}H NMR (500 MHz, $CDCl_3$, δ ppm, 25 °C, **Figure S14**): 8.91 (s, 2H), 8.76 (s, 2H), 7.18 (s, 4H), 7.38–7.29 (br, 2H), 7.24–7.16 (br, 2H) 4.26 (s, 4H), 1.57 (s, 2H), 1.42–0.75 (br, 154H). M_n (GPC) = 5,950, PDI = 1.4 (bimodal molecular weight distribution due to the polymer aggregates). Anal. Calcd for $C_{118}H_{178}N_2O_{12}S_2$ (%): C, 75.4; H, 9.5; N, 1.5; S, 3.4. Found (%): C, 80.0; H, 11.1; N, 0.7; S, 1.4. Yield = 67% (130 mg).

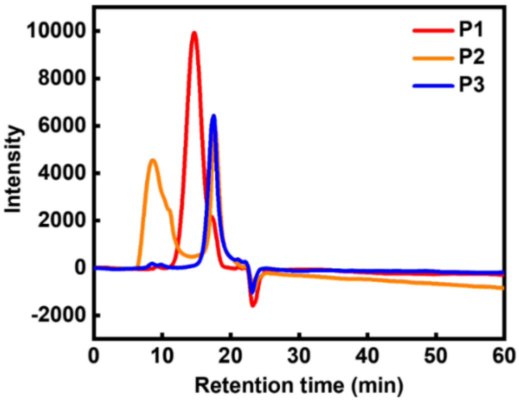

Figure S1. GPC profiles of the CPs in THF at 40 °C and 0.3 mL min^{-1}. Note that the molecular weight used poly(methyl methacrylate) (PMMA) as the molecular weight standard.

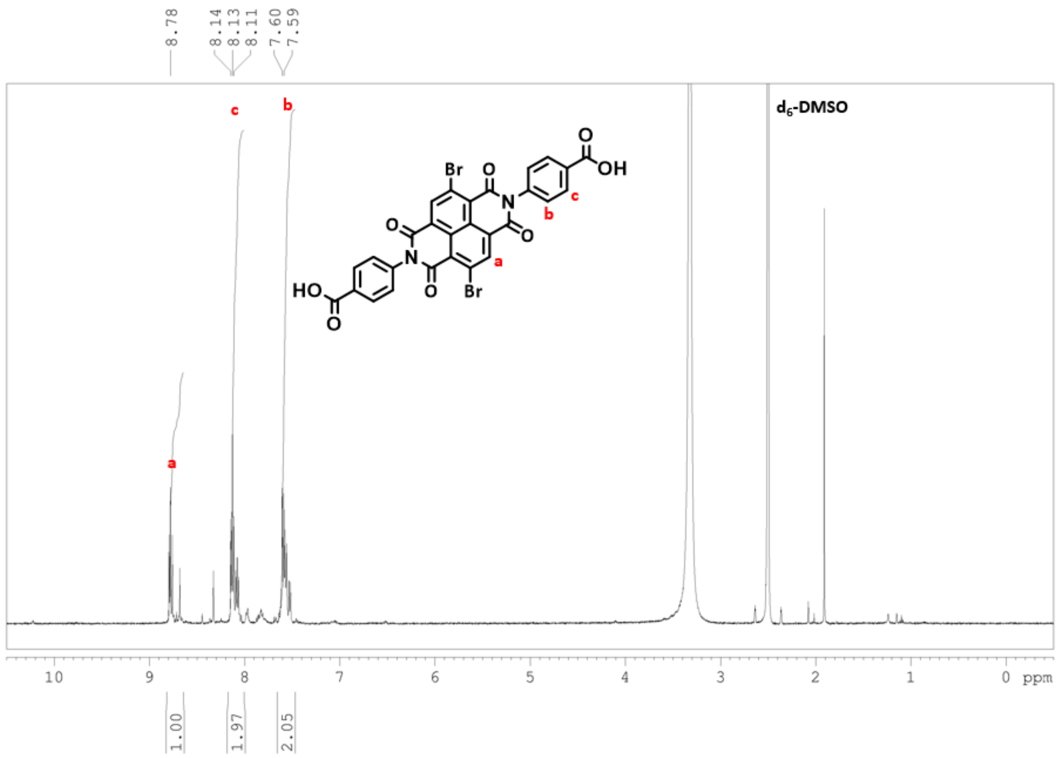

Figure S2. ^{1}H NMR spectrum of **M1** in DMSO-d_6.

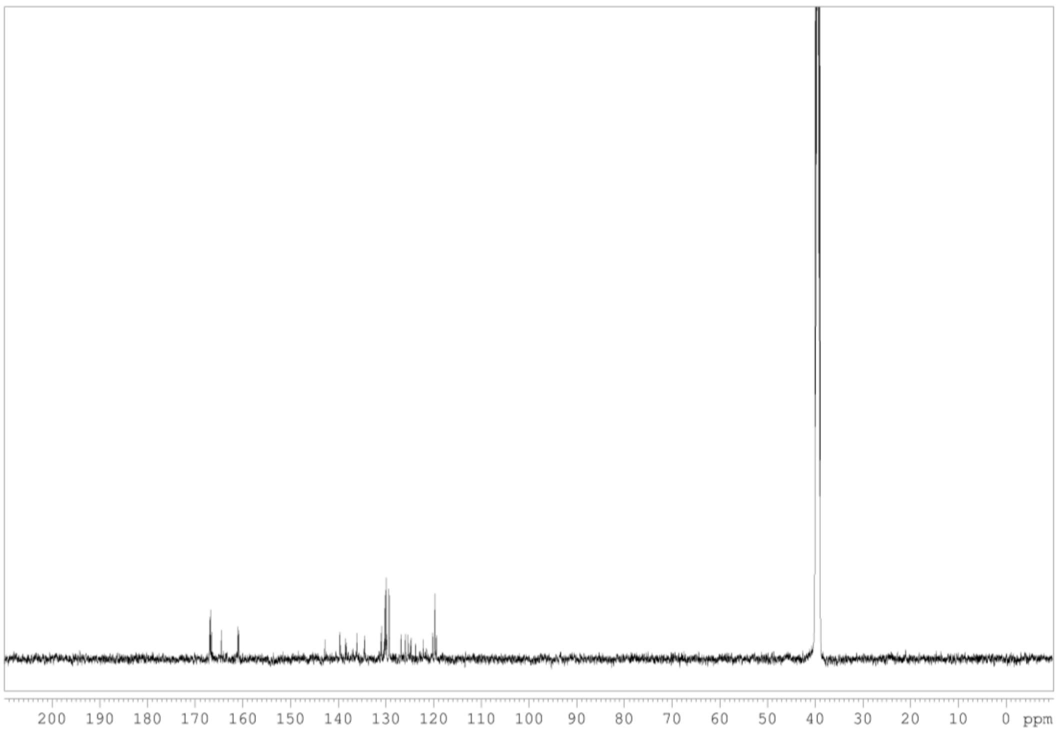

Figure S3. ^{13}C NMR spectrum of **M1** in DMSO-d_6.

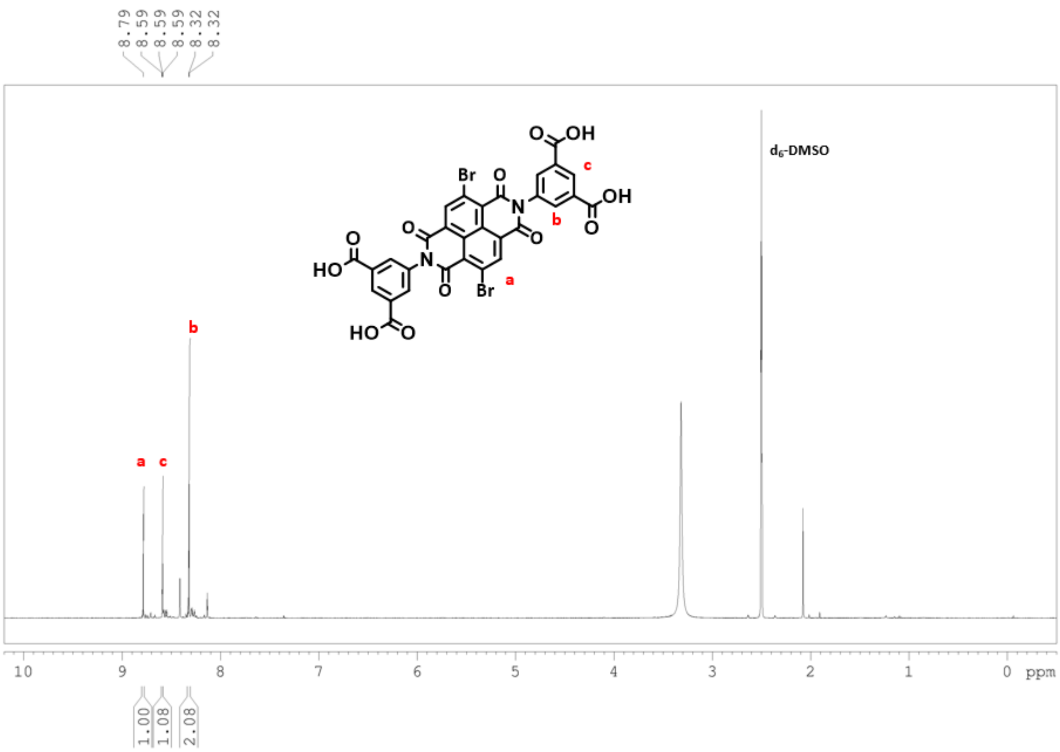

Figure S4. ^{1}H NMR spectrum of **M2** in DMSO-d_6.

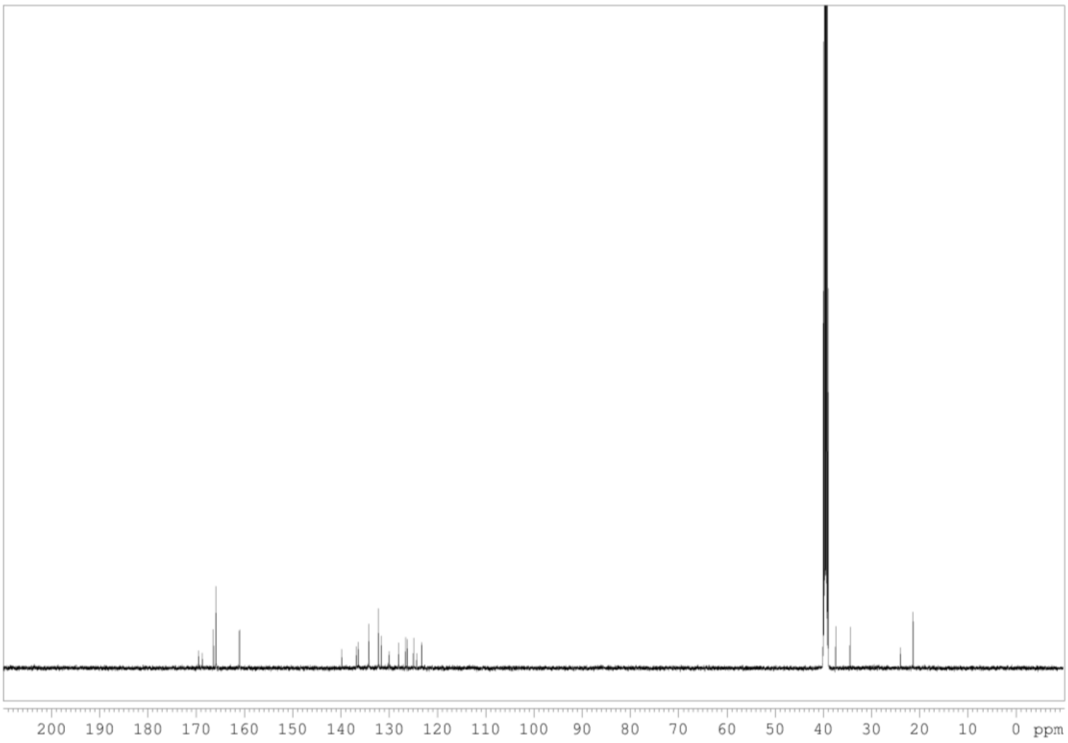

Figure S5. ^{13}C NMR spectrum of **M2** in DMSO-d_6.

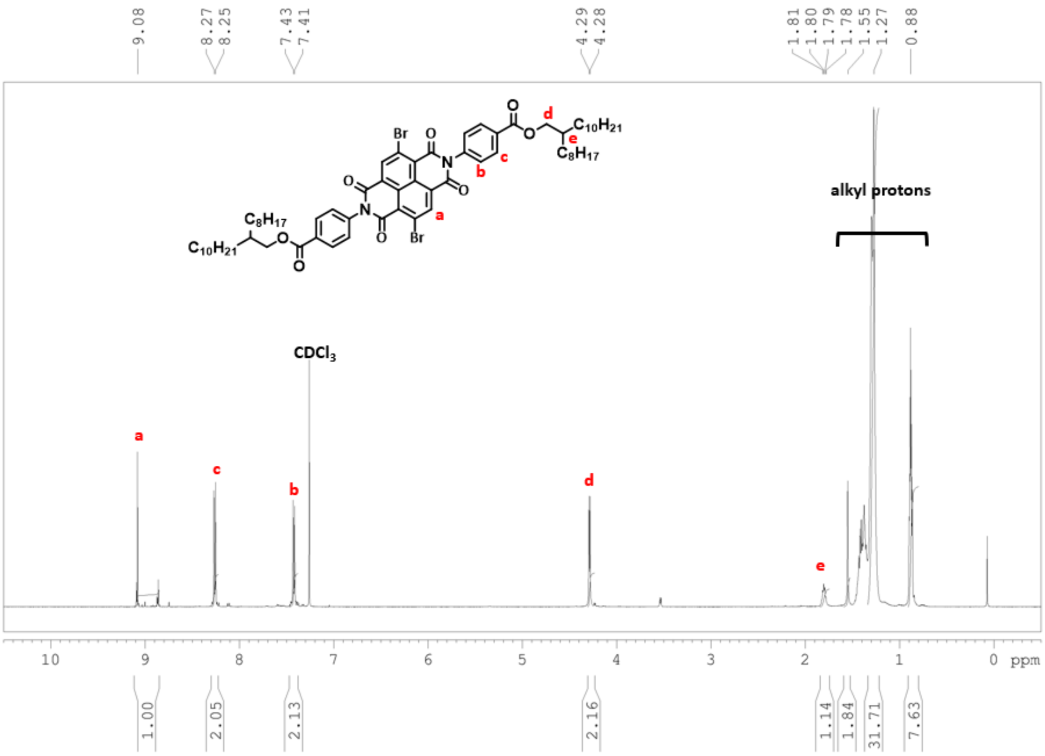

Figure S6. ^{1}H NMR spectrum of **M3** in $CDCl_3$.

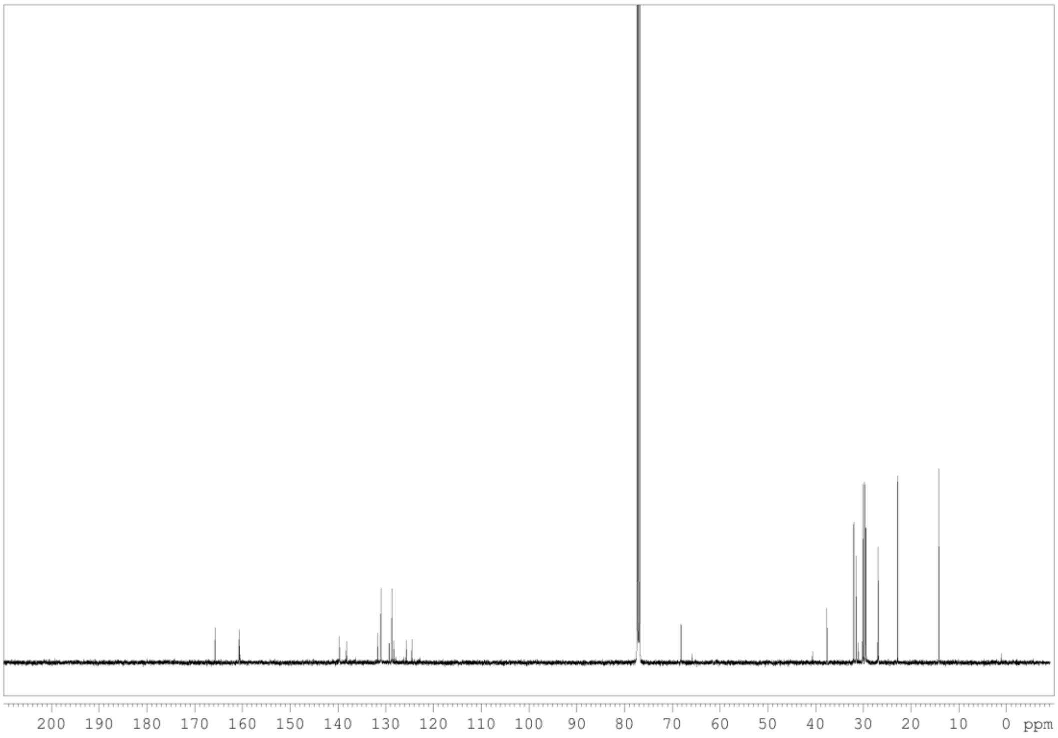

Figure S7. ^{13}C NMR spectrum of **M3** in $CDCl_3$.

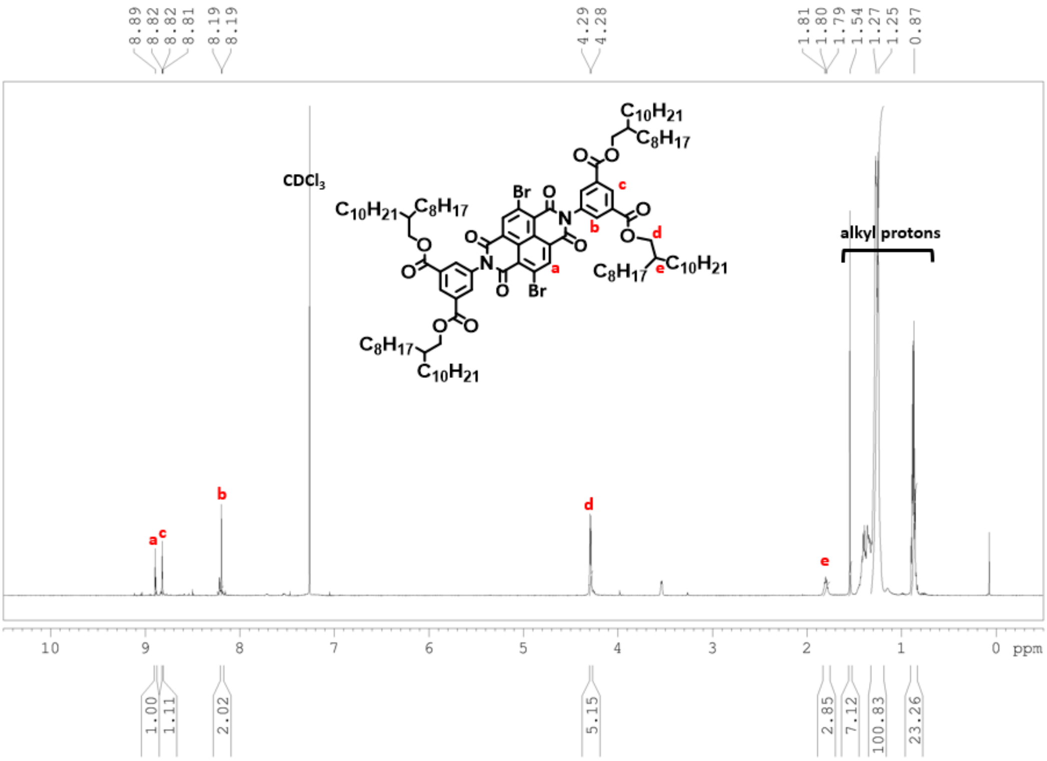

Figure S8. ^{1}H NMR spectrum of **M4** in $CDCl_3$.

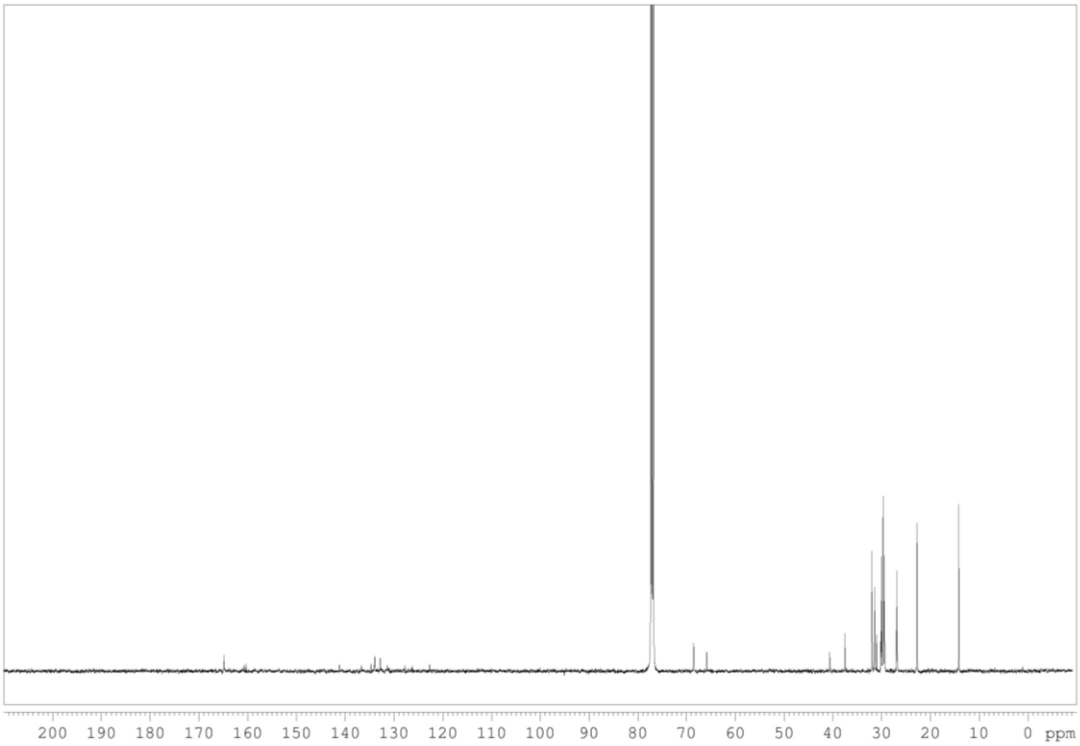

Figure S9. ^{13}C NMR spectrum of **M4** in $CDCl_3$.

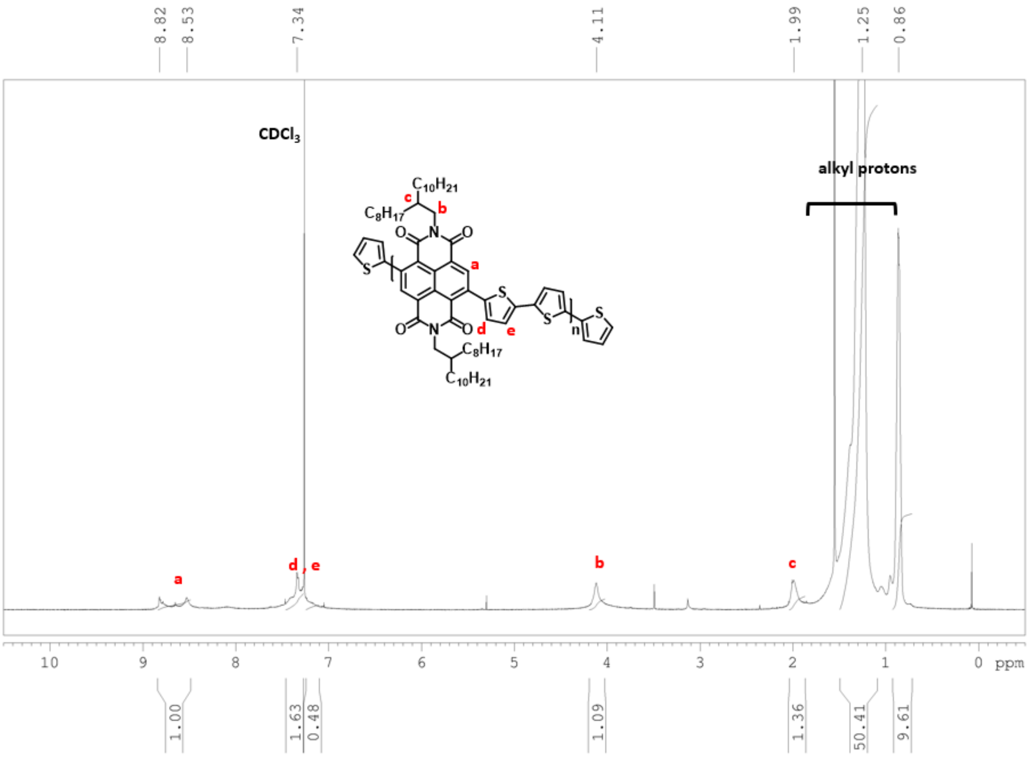

Figure S10. ^{1}H NMR spectrum of **P1** in $CDCl_3$.

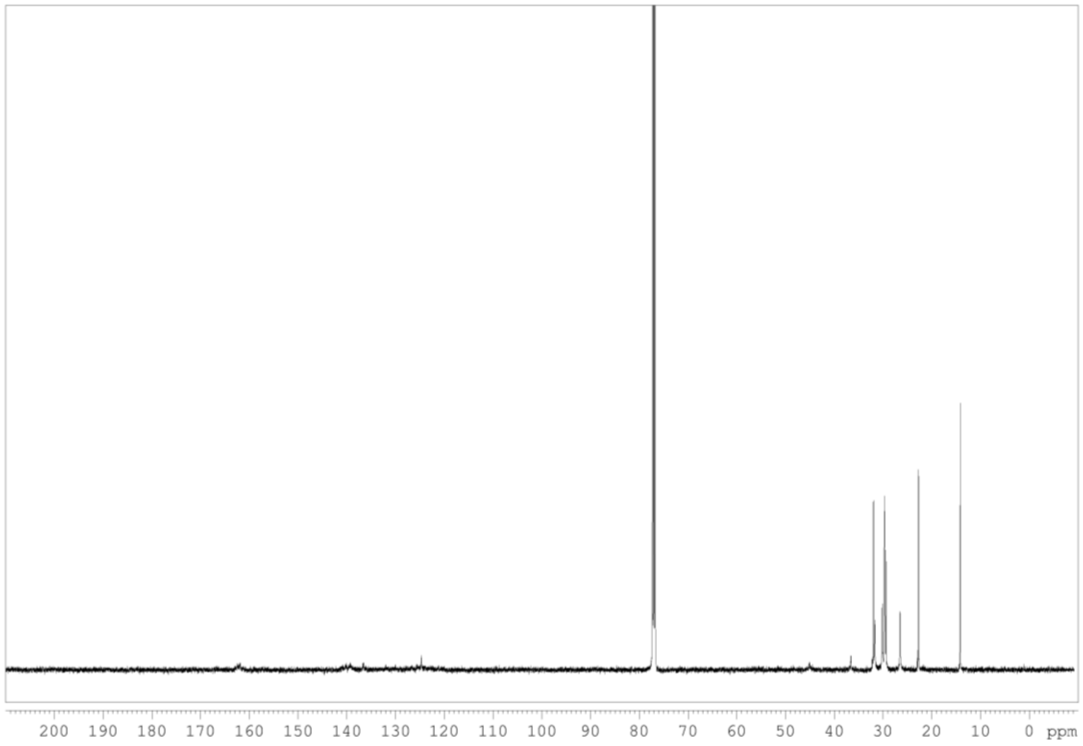

Figure S11. ^{13}C NMR spectrum of **P1** in $CDCl_3$.

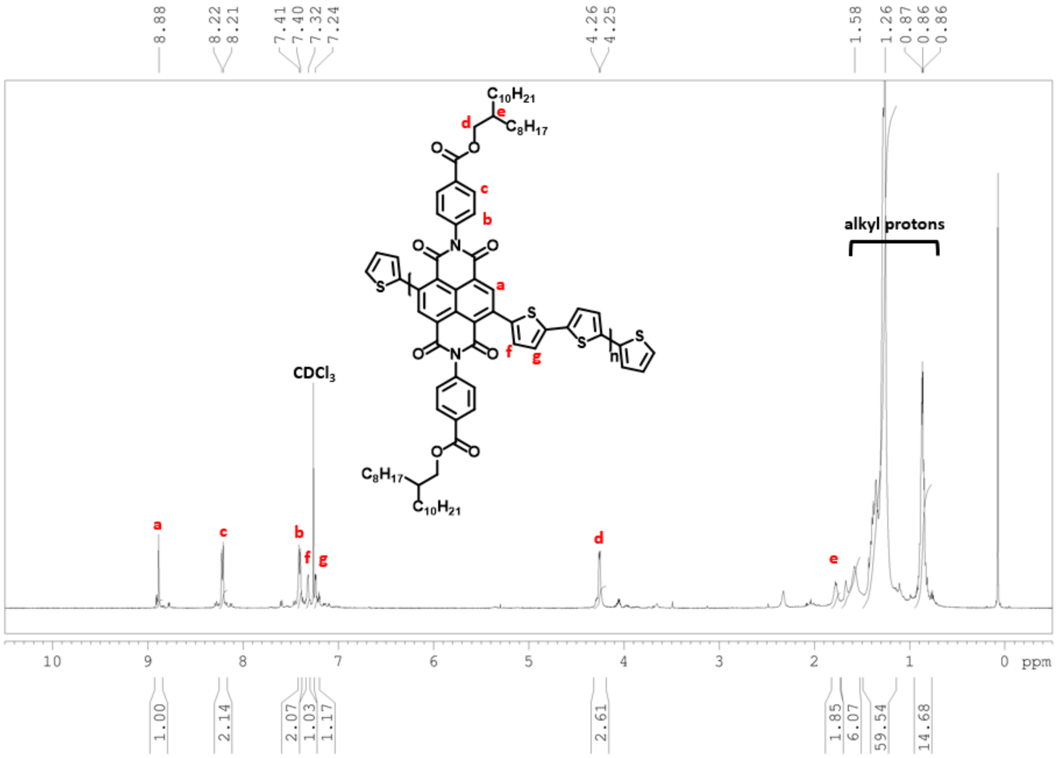

Figure S12. ^{1}H NMR spectrum of **P2** in $CDCl_3$.

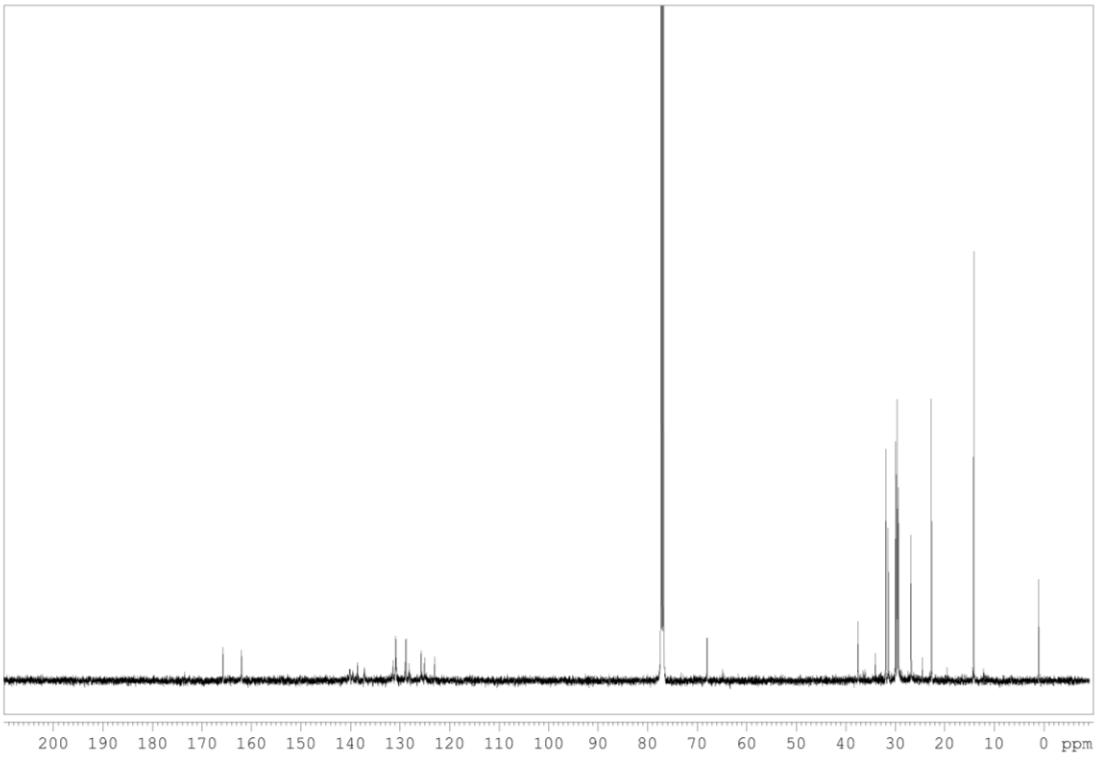

Figure S13. ^{13}C NMR spectrum of **P2** in $CDCl_3$.

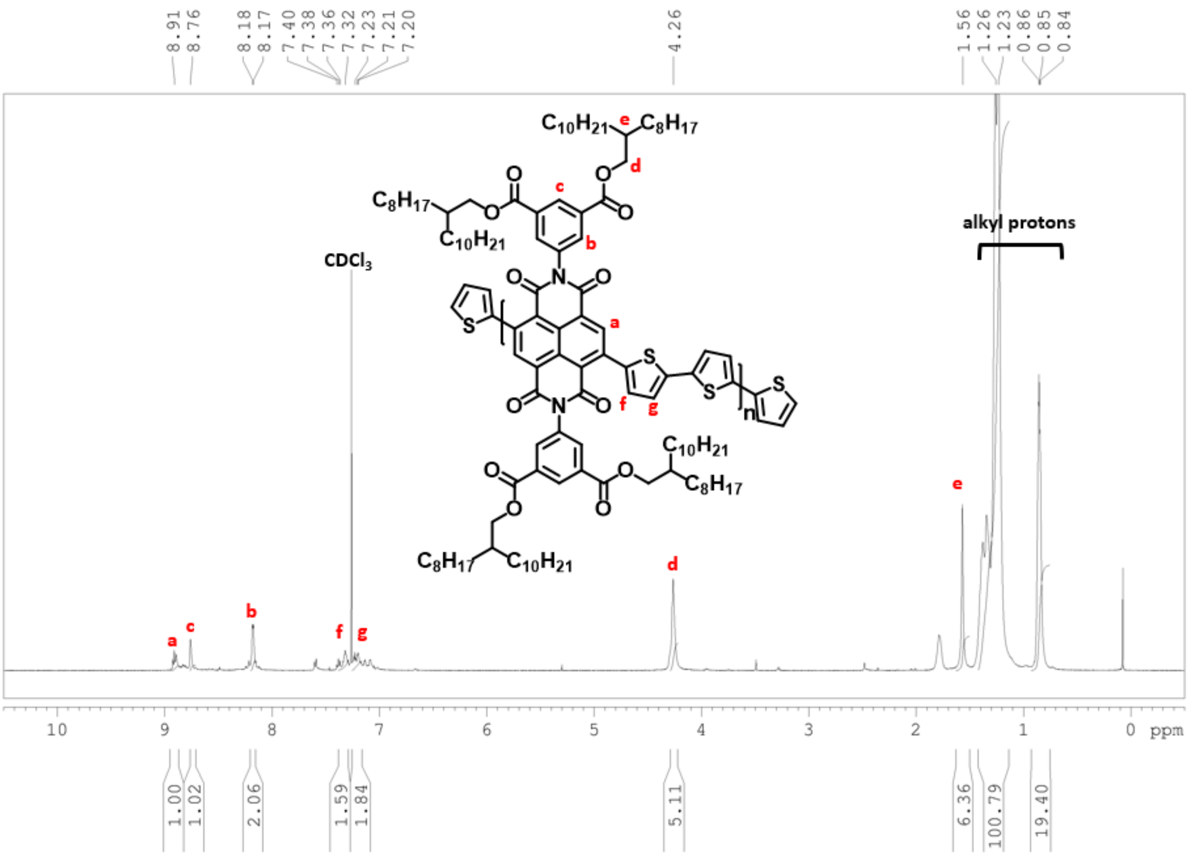

Figure S14. ^{1}H NMR spectrum of **P3** in $CDCl_3$.

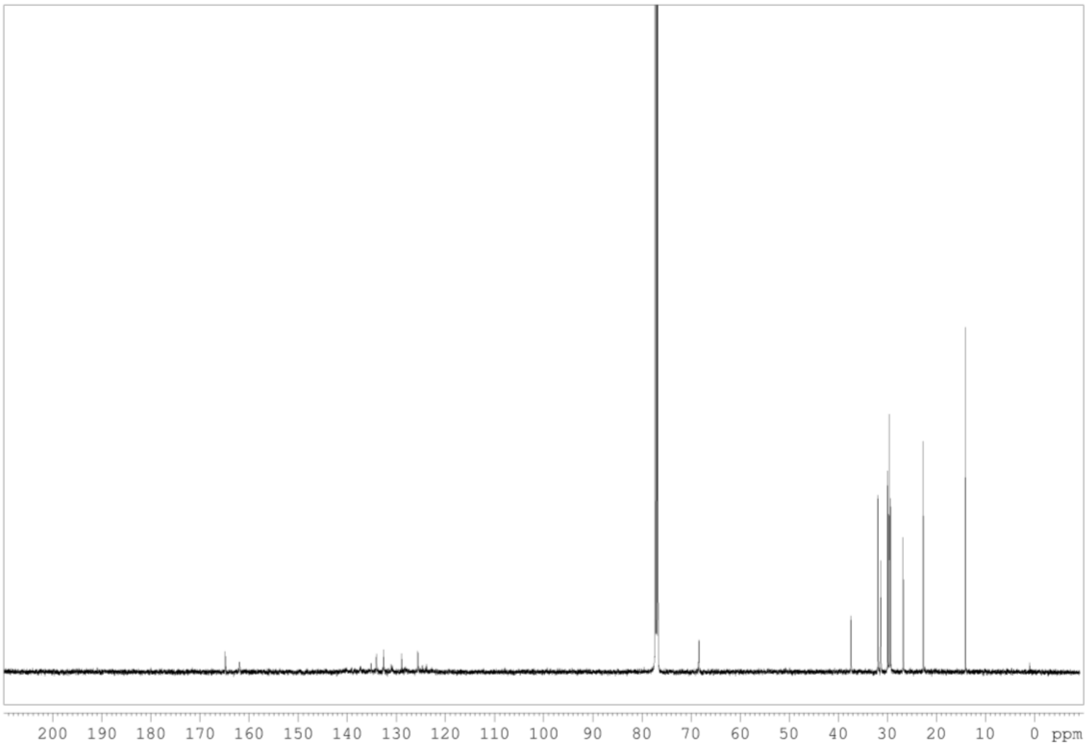

Figure S15. ^{13}C NMR spectrum of **P3** in $CDCl_3$.

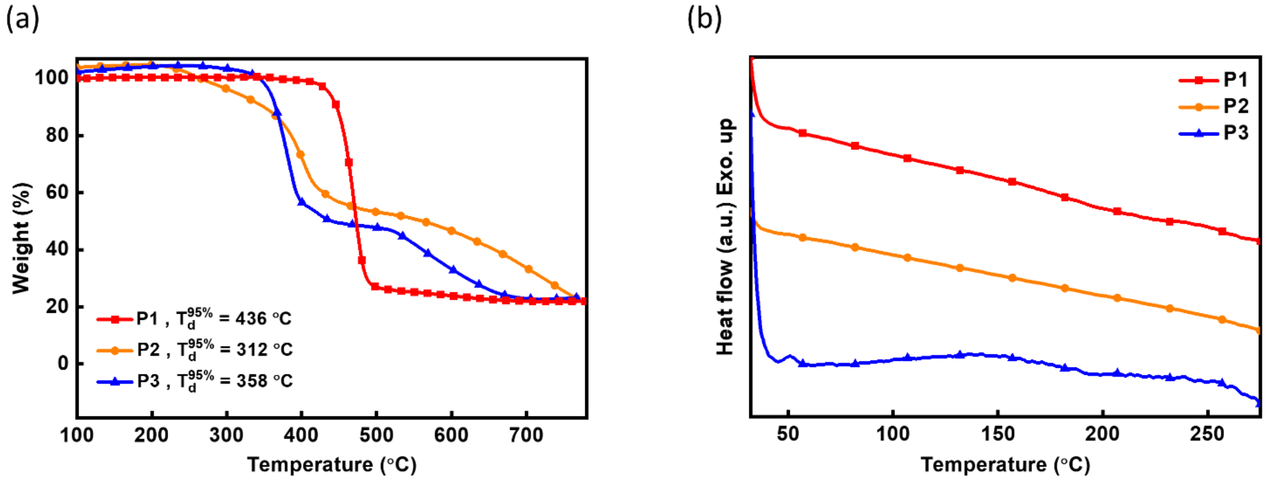

Figure S16. Thermal properties of the polymers studied: (a) TGA and (b) DSC profiles at a ramping rate of 10 °C min^{-1}.

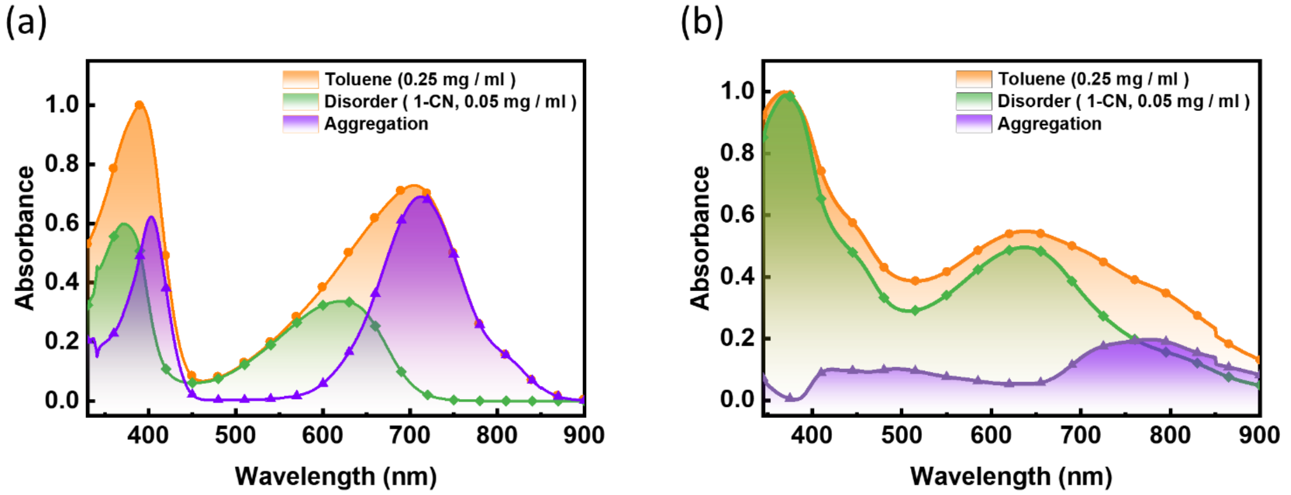

Figure S17. Aggregation and disorder fractions in the UV–Vis absorption spectra of polymers (a) **P1** and (b) **P2**. Note that the polymer solutions in toluene were prepared at a concentration of 0.25 mg mL^{-1}, and the disordered polymer solutions were prepared in 1-chloronaphthalene (1-CN) at a concentration of 0.05 mg mL^{-1}.

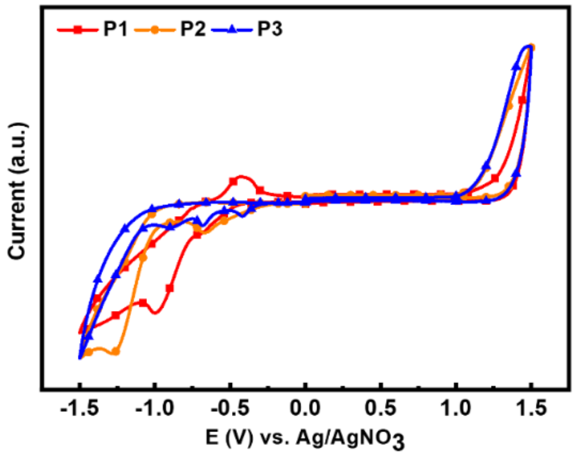

Figure S18. CV profiles of the polymer films coated on an ITO glass. The measurement was conducted at a scanning rate of 0.1 V s^{-1}.

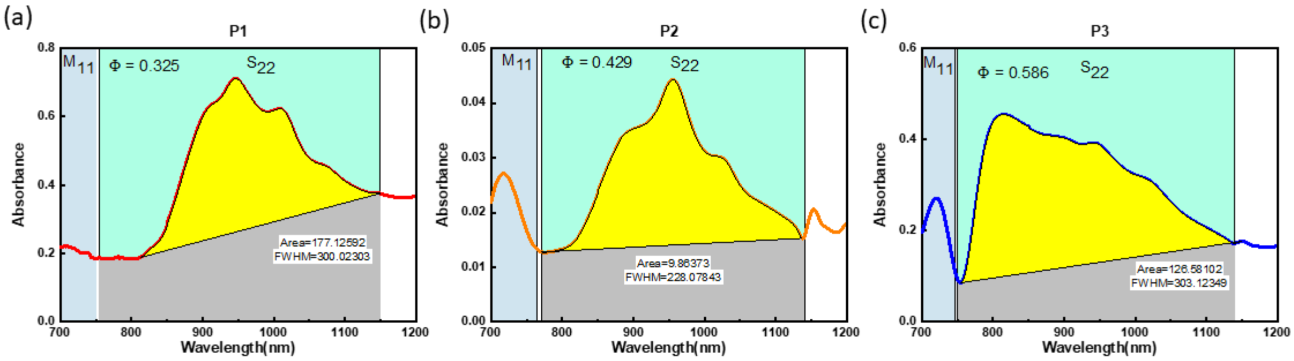

Figure S19. UV–Vis absorption spectra of the *sc*-SWNTs sorting solutions for calculating its purity: (a) **P1**/*sc*-SWNT, (b) **P2**/*sc*-SWNTs, and (c) **P3**/*sc*-SWNTs. The absorbances of NDI-based CPs were deconvoluted and subtracted from the absorption spectra of CP/*sc*-SWNT solutions.

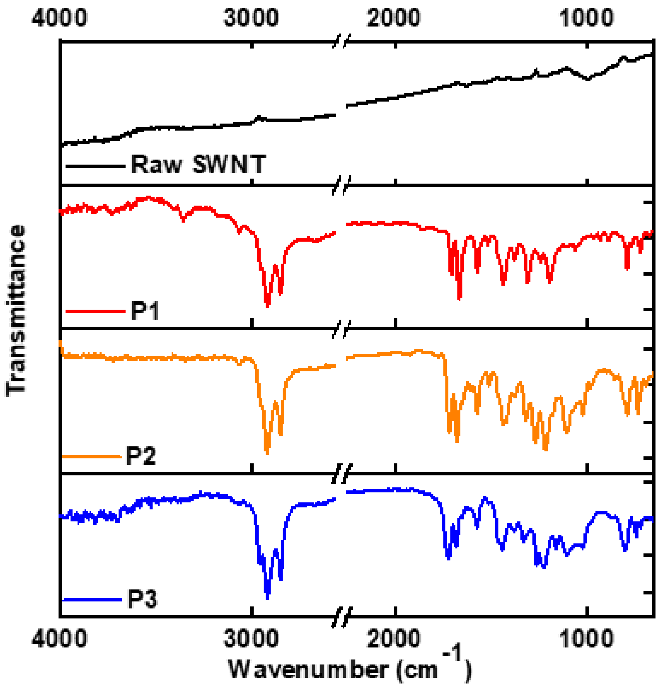

Figure S20. FT-IR spectra of the raw SWNT, and pure polymer powder of **P1**, **P2**, and **P3**.

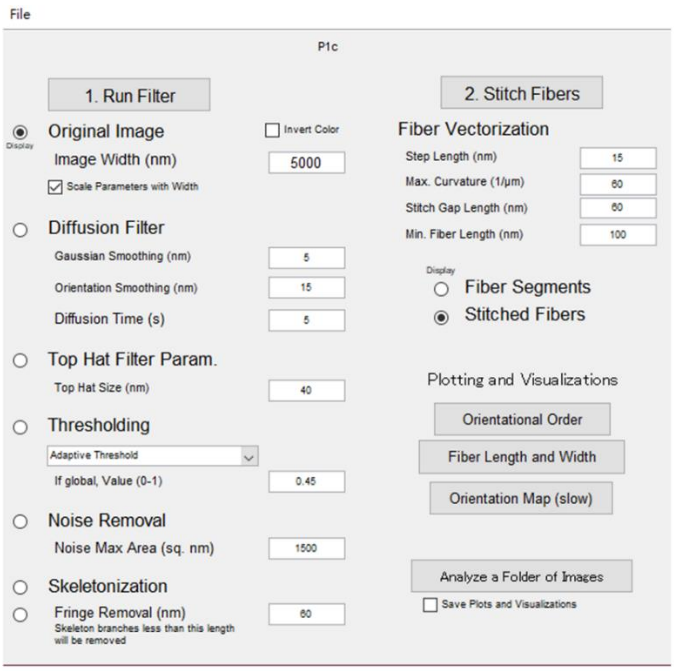

Figure S21. SWNT morphology fitting parameters in GTFiber software for extracting the lengths of *sc*-SWNTs in AFM topography. The program was developed by Persson *et al.* and reported in *Chem. Mater.* **2017**, *29*, 3-14.

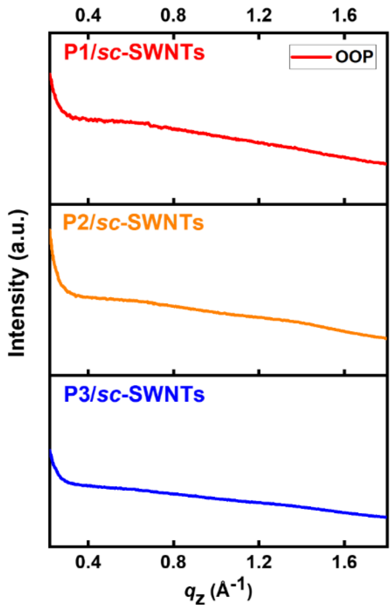

Figure S22. 1D GIXD profiles of CP/*sc*-SWNT films extracted along the OOP direction: the lamellar stacking is inhibited with the existence of *sc*-SWNTs.

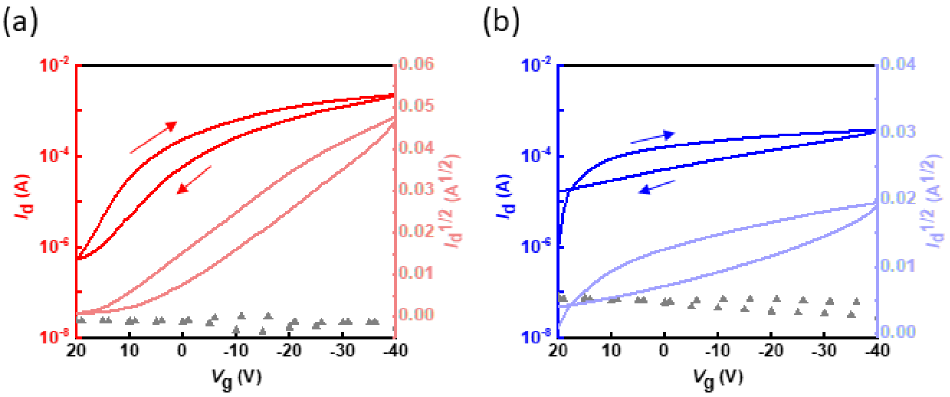

Figure S23. Transfer characteristics curves with V_d = –100 V for CP/*sc*-SWNT devices of (a) **P1**/*sc*-SWNTs and (b) **P3**/*sc*-SWNTs. Note that gray scattered dots represent the gate current measured, and the curves were swept from 20 to –40 V for *p*-type operation.

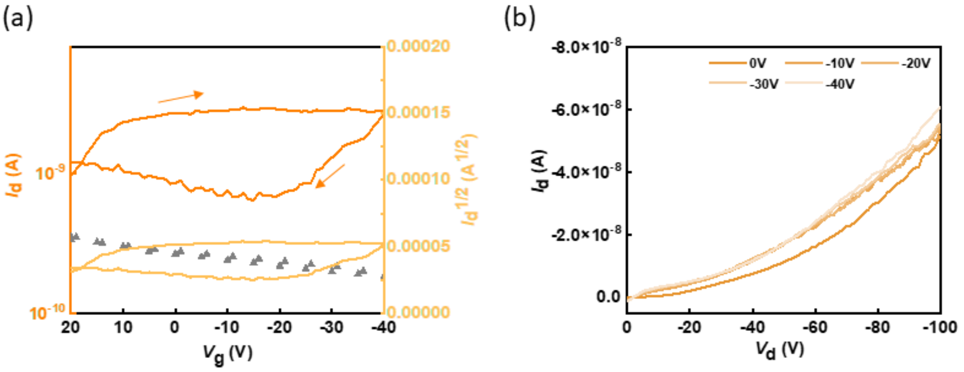

Figure S24. (a) Transfer curve with V_d = −10 V and (b) output curve of **P2**/*sc*-SWNTs device. Note that the device's drain current is low due to the low yield of *sc*-SWNTs sorted by **P2**.

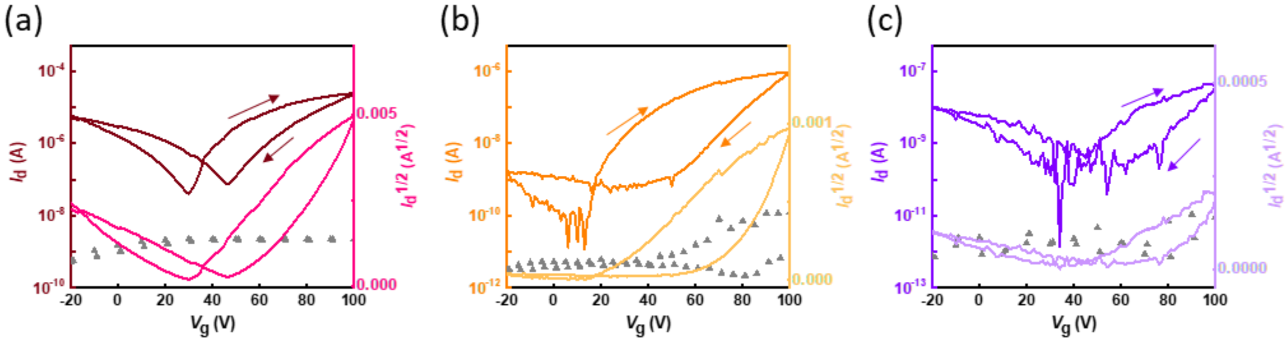

Figure S25. FET transfer characteristics of the *n*-type CP films of (a) **P1**, (b) **P2**, and (c) **P3**. Note that the gray scattered dots represent the gate currents measured, and the curves were swept from –20 to 100 V for *n*-type operation.

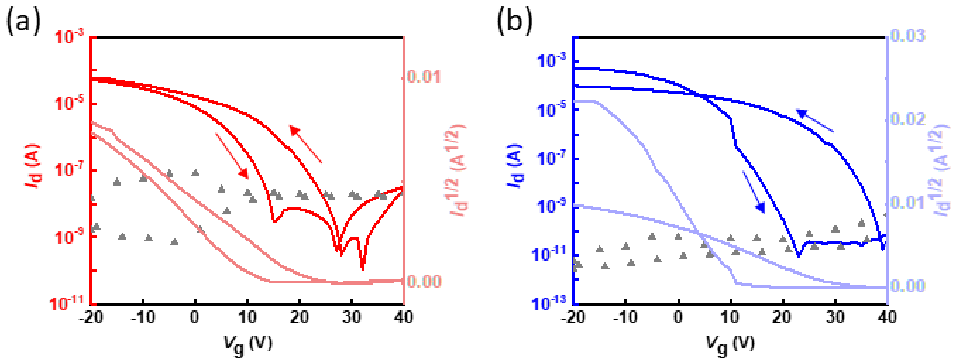

Figure S26. N-type transfer characteristic curves of the CP/*sc*-SWNTs device of (a) **P1**/*sc*-SWNTs and (b) **P3**/*sc*-SWNTs. Note that the curves were swept from −20 to 40 V at V_d = 10 V.

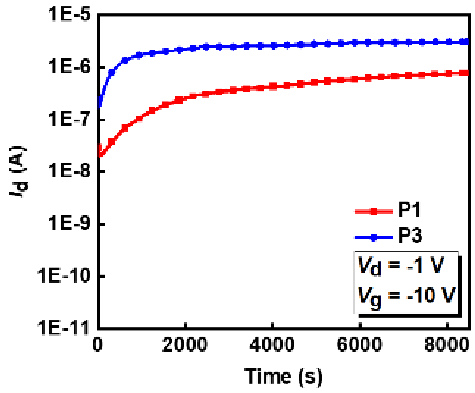

Figure S27. Bias stability test of CP/*sc*-SWNT based devices by applying $V_g = -10$ V at $V_d = -1$ V.

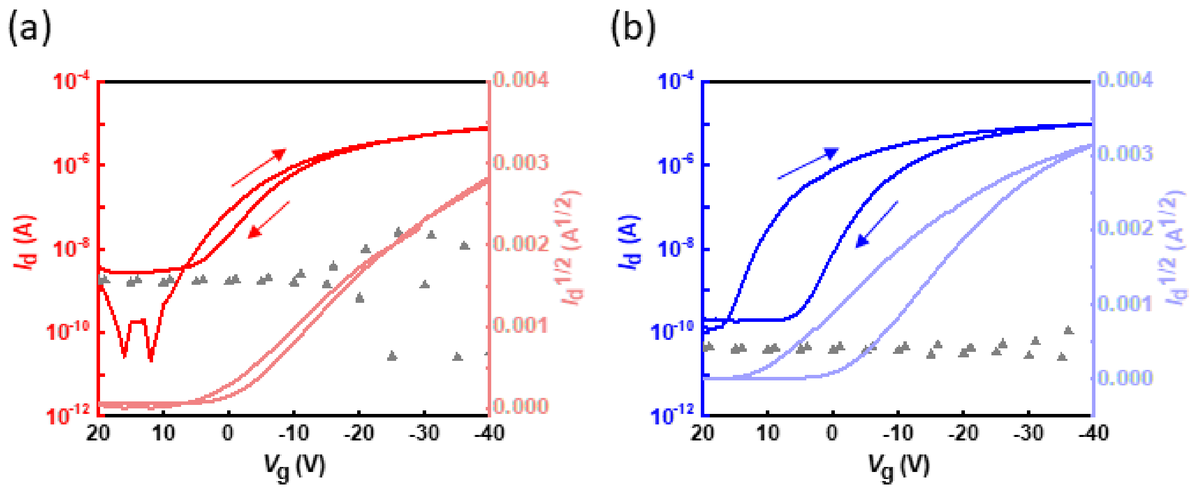

Figure S28. Transfer characteristics curves with $V_d = -1$ V for the CP/*sc*-SWNT devices of (a) **P1**/*sc*-SWNTs and (b) **P3**/*sc*-SWNTs. Note that gray scattered dots represent the gate current measured, and the curves were swept from 20 to −40 V for *p*-type operation.
